# Biomarker discovery in Alzheimer's and neurodegenerative diseases using Nucleic Acid Linked Immuno‐Sandwich Assay

**DOI:** 10.1002/alz.14621

**Published:** 2025-05-22

**Authors:** Nicholas J. Ashton, Andrea L. Benedet, Guglielmo Di Molfetta, Ilaria Pola, Federica Anastasi, Aida Fernández‐Lebrero, Albert Puig‐Pijoan, Ashvini Keshavan, Jonathan Schott, Kubra Tan, Joel Simrén, Bárbara Fernandes Gomes, Laia Montoliu‐Gaya, Richard Isaacson, Matilde Bongianni, Chiara Tolassi, Valentina Cantoni, Antonella Alberici, Alessandro Padovani, Gianluigi Zanusso, Andrea Pilotto, Barbara Borroni, Marc Suárez‐Calvet, Kaj Blennow, Henrik Zetterberg

**Affiliations:** ^1^ Department of Psychiatry and Neurochemistry Institute of Neuroscience & Physiology the Sahlgrenska Academy at the University of Gothenburg Mölndal Sweden; ^2^ Banner Alzheimer's Institute and University of Arizona Phoenix Arizona USA; ^3^ Banner Sun Health Research Institute Sun City Arizona USA; ^4^ Hospital del Mar Research Institute Barcelona Spain; ^5^ Barcelonaβeta Brain Research Center (BBRC) Pasqual Maragall Foundation Barcelona Spain; ^6^ Servei de Neurologia Hospital del Mar Barcelona Spain; ^7^ Department of Medicine and Life Sciences Universitat Pompeu Fabra Barcelona Spain; ^8^ Department of Medicine Universitat Autònoma de Barcelona Barcelona Spain; ^9^ Dementia Research Centre UCL Queen Square Institute of Neurology University College London London UK; ^10^ UK Dementia Research Institute at UCL London UK; ^11^ Department of Neurology Weill Cornell Medicine and New York Presbyterian New York New York USA; ^12^ Department of Neurology Florida Atlantic University, Charles E. Schmidt College of Medicine Boca Raton Florida USA; ^13^ Department of Neurosciences, Biomedicine, and Movement Sciences Policlinico G. B. Rossi, University of Verona Verona Italy; ^14^ Clinical Investigation in Laboratory Maggiore Hospital ASST‐Crema Crema Italy; ^15^ Department of Clinical and Experimental Sciences, Neurology Unit University of Brescia Brescia Italy; ^16^ Department of Continuity of Care and Frailty Azienda Socio Sanitaria Territoriale (ASST) Spedali Civili Brescia Italy; ^17^ Laboratory of Digital Neurology and Biosensors University of Brescia Brescia Italy; ^18^ Brain Health Center University of Brescia Brescia Italy; ^19^ Department of Neuroscience, Biomedicine and Movement Sciences University of Verona Verona Italy; ^20^ Centro de Investigación Biomédica en Red de Fragilidad y Envejecimiento Saludable (CIBERFES) Madrid Spain; ^21^ Clinical Neurochemistry Laboratory Sahlgrenska University Hospital Mölndal Sweden; ^22^ Paris Brain Institute ICM, Pitié‐Salpêtrière Hospital, Sorbonne University, Hôpital Pitié Paris France; ^23^ Neurodegenerative Disorder Research Center Division of Life Sciences and Medicine and Department of Neurology Institute on Aging and Brain Disorders University of Science and Technology of China and First Affiliated Hospital of USTC Hefei P. R. China; ^24^ Hong Kong Center for Neurodegenerative Diseases Hong Kong China; ^25^ Wisconsin Alzheimer's Disease Research Center University of Wisconsin School of Medicine and Public Health, University of Wisconsin–Madison Madison Wisconsin USA

**Keywords:** discovery, frontotemporal dementia, Lewy body disease, Nucleic Acid Linked Immuno‐Sandwich Assay, plasma biomarkers, proteomics

## Abstract

**INTRODUCTION:**

Recent advancements in immunological methods accurately quantify biofluid biomarkers for Alzheimer's disease (AD) pathology. Despite progress, more biomarkers, ideally in blood, are needed for effective disease monitoring for AD and other neurodegenerative proteinopathies.

**METHODS:**

We used the Nucleic Acid Linked Immuno‐Sandwich Assay (NULISA) central nervous system panel for biomarker quantification in plasma, serum, and cerebrospinal fluid of patients with AD, mild cognitive impairment, Lewy body dementia, progranulin (*GRN*) mutation carriers.

**RESULTS:**

NULISA identified phosphorylated tau217 and neurofilament light chain as the most deregulated biomarkers in the AD continuum and *GRN* mutation carriers, respectively. Importantly, numerous novel proteomic changes were observed in each disease endophenotype, which included synaptic processing, inflammation, microglial reactivity, TAR DNA‐binding protein 43, and α‐synuclein pathology.

**DISCUSSION:**

We underline the potential of next‐generation biomarker identification tools to detect novel proteomic features that also incorporate established biomarkers. These findings highlight the importance of continued biomarker discovery to improve treatment decisions and help us better understand the complexities of neurodegenerative disorders.

**Highlights:**

- The, direct, or indirect, measures in blood that complement phosphorylated tau (p‐tau)217 for other proteinopathies or disease progression are urgently needed.
- Significant novel proteomic changes were observed in each disease endophenotype in plasma, serum, and cerebrospinal fluid, which included proteins involved in synaptic processing, inflammation, microglial reactivity, TAR DNA‐binding protein 43, and α‐synuclein pathology.
- Nucleic Acid Linked Immuno‐Sandwich Assay continued to unbiasely highlight p‐tau217 and neurofilament light chain as the most significantly deregulated blood biomarkers in the Alzheimer's disease continuum and progranulin mutation carriers, respectively.

## 1 BACKGROUND

Discovery plasma proteomics has long since demonstrated a differential signal in the blood of patients clinically diagnosed with Alzheimer's disease (AD).^1^, ^2^ These earlier studies used agnostic methods to discover novel biomarkers—proteomic technologies not predicated on any a priori hypotheses in a case‐control approach^1^, ^3^, ^4^, ^5^, ^6^ but later evolved to an endophenotype design based on pathology; brain atrophy measures,^1^, ^7^, ^8^, ^9^ cerebrospinal fluid (CSF) biomarkers,^10^ or amyloid beta (Aβ) positron emission tomography (PET).^11^, ^12^, ^13^, ^14^, ^15^ These reports continually implicated a dysregulated complement, acute inflammation response,^5^, ^16^ and markers previously identified in genome‐wide association studies (e.g., clusterin^17^) but failed in identifying a replicable biomarker signature with the required disease specificity or clinical usefulness.

Advancements in ultra‐sensitive immunoassays and highly specific immunoprecipitation mass spectrometry methods, complemented by an increasing number of well‐characterized research cohorts that adhere to a biological definition of disease,^18^ have substantially contributed to the rapid development of key targets in blood. In Ad, measures of phosphorylated tau (p‐tau), particularly p‐tau217,^19^, ^20^ have shown utility in identifying cerebral pathology at all stages of the disease continuum. Thus, it is anticipated that plasma p‐tau217 will be an essential component of patient management and treatment decisions related to AD. As a disease‐defining biomarker in CSF, the detection of Aβ42/40 in plasma has seen much improvement,^21^, ^22^ and while it is unlikely to feature as a primary diagnostic blood biomarker, due to several confounding factors (e.g., poor robustness,^23^ pharmacodynamic effects,^24^ and pre‐analytical instability), it will have relevance in understanding novel disease‐modifying therapies (DMTs) that shift Aβ production to a less pathogenic state.

Yet, there remains a multitude of biomarker applications in AD and related disorders in which plasma p‐tau217 and Aβ42/40 are not fully sufficient. These blood biomarkers do not entirely explain the extent of tau accumulation,^25^ longitudinal clinical progression,^26^ or response to amyloid clearance,^27^ although p‐tau217 is the leading candidate of such associations. In the light of approved DMTs, prognostic biomarker signatures of intervention response or risk to adverse events are greatly needed to guide treatment management prior to administration. Further, p‐tau217 and Aβ42/40 only work to identify AD pathology as the main or contributing factor in other neurogenerative proteinopathies. AD accounts for ≈ 60% of all diagnosed dementia cases, and supplemental biomarkers are required to differentiate other forms of dementia to further improve patient management and accelerate drug development. Promising results from α‐synuclein (αSyn) seed amplification assays (SAAs) in Lewy body (LB) dementia have not yet translated from CSF to blood, and biomarkers of vascular, TAR DNA‐binding protein 43 (TDP‐43) and 4R tau pathologies remain unresolved. In blood, neurofilament light chain (NfL) shows limited disease specificity,^28^ with some isolated instances, for example, atypical parkinsonian disorders compared to Parkinson's disease,^29^ but remains a useful biomarker for general neurodegeneration and acute neurological injury. Glial fibrillary acidic protein (GFAP) in blood has also shown changes in some non‐AD pathologies^30^, ^31^ but recent data have clearly demonstrated its dynamic change in response to amyloid and tau deposition.^32^, ^33^, ^34^, ^35^ Thus, direct, or indirect, measures in blood that are more specific to other proteinopathies are urgently needed.

Proteomic studies of biofluid biomarkers can be targeted or non‐targeted; the latter often guiding the former. A common non‐targeted approach to identify blood‐based biomarkers is liquid chromatography tandem mass spectrometry (LC–MS/MS) but it has limited coverage of the plasma proteome due to signals derived from low‐abundant proteins often masked by those of higher concentrations, thus making LC‐MS/MS not optimal for the detection at the sub‐picomolar level.^11^, ^12^ Instead, high‐plex solutions with attomolar sensitivity, such as Olink, SomaLogic, and now Nucleic Acid Linked Immuno‐Sandwich Assay (NULISA), can offer targeted methods with hundreds to thousands of proteins in low volume and simplified workflows.

**RESEARCH IN CONTEXT**

1. **Systematic review**: The authors reviewed the literature using traditional (e.g., PubMed) sources. While there are currently no published studies using the Nucleic Acid Linked Immuno‐Sandwich Assay (NULISA) central nervous system ^(^CNS) panel in biomarker research, several studies have used multi‐analyte technologies to describe the blood biomarker profiles in neurodegenerative disease endophenotypes described in this paper.
2. **Interpretation**: Our findings demonstrate that the NULISA CNS panel is a useful new tool for identifying novel biomarkers and biomarker patterns in the neurodegenerative disease area, addressing a currently unmet need. The NULISA CNS panel also incorporates a high‐performance version of established biomarkers (e.g., phosphorylated tau [p‐tau]217, glial fibrillary acidic protein [GFAP], and neurofilament light chain [NfL]).
3. **Future directions**: We highlight the potential of next‐generation biomarker identification tools, such as NULISA, to detect novel proteomic features that incorporate established biomarkers like p‐tau217 and NfL. This collection of pilot study highlights needs to be replicated in larger sample sizes to affirm the novel markers suggested; however, the independent and unbiased validation of p‐tau217, GFAP, and NfL enhances the credibility of these novel findings.

This collection of pilot studies aims to provide an example in a new direction of biomarker evaluation and discovery. This would be to combine high‐precision measurements of known biomarkers of neurodegenerative disease (e.g., p‐tau217, GFAP, and NfL) within a multi‐analyte panel for high‐throughput analysis. This study uses the NULISA central nervous system (CNS) panel in four cohorts with different examine endophenotype profiles.

## 2 METHODS

### 2.1 Participants and ethics

In cohort 1, 40 participants were recruited from the prospective University College London Dementia Research Centre as a part of the Alzheimer's Association's Global Biomarker Standardization Consortium (GBSC) plasma phospho‐tau Round Robin study. Participants, who were under clinical evaluation for cognitive impairment, were classified according to their CSF Aβ42/40 and p‐tau181 (Lumipulse G; Fujirebio) concentrations. Participants were confirmed as AD pathology if CSF A β 42/40 was < 0.065.^36^ All participants gave written informed consent according to the Declaration of Helsinki. The study was approved by the local ethics committee (Wolfson CSF study 12/0344). In cohort 2, 40 participants were selected from the BIODEGMAR cohort, an observational longitudinal study that enrolls individuals with cognitive symptoms and/or neurodegenerative diseases visiting the Cognitive and Behavioral Unit of Hospital del Mar (Barcelona, Spain).^37^ Specifically, all selected participants in this study had a diagnosis of mild cognitive impairment (MCI).^38^ Core AD CSF biomarkers (Aβ42/40, p‐tau, and total tau [t‐tau]) were measured with Lumipulse immunoassays (Fujirebio). Participants were classified as AD CSF profile if the CSF Aβ42/p‐tau ratio was < 10.25.^37^ In cohort 3, 54 participants were enrolled at the Neurology Unit of Brescia. The presence or absence of LB (LB ±) pathology was determined by αSyn SAA at the University of Verona as previously reported.^39^ The LB– patients met the National Institute on Aging–Alzheimer's Association clinical criteria for the diagnosis of AD (*n* = 30) confirmed based on a CSF biomarker profile (p‐tau/Aβ42 ratio > 0.9).^40^ The LB+ patients were clinically classified as dementia with Lewy bodies (DLB; *n* = 22), according to the definitions and guidelines provided by the DLB Consortium, and Parkinson's disease (PD; *n* = 2) according to current clinical criteria.^41^ LB+ patients were negative for AD biomarkers. All participants gave written informed consent according to the Declaration of Helsinki. The study was approved by the local ethics committee (NP 1471). In cohort 4, 40 participants were enrolled at the Center for Neurodegenerative Disorders, University of Brescia (Italy) from October 3, 2023, to November 23, 2023. In frontotemporal dementia (FTD) patients (*n* = 18), the presence pathogenetic progranulin (*GRN*) mutations was carried out according to standard procedures.^42^ Presymptomatic *GRN* mutation carriers (*n* = 2)^43^ and healthy controls (HCs, *n* = 20), recruited among spouses or patients’ family members, were also included. The FTD patients met current clinical criteria for the diagnosis of agrammatic variant of primary progressive aphasia (avPPA, *n* = 11), behavioral variant frontotemporal dementia (bvFTD, *n* = 6), semantic variant of primary progressive aphasia (svPPA, *n* = 1). All participants gave written informed consent according to the Declaration of Helsinki. The study was approved by the local ethics committee (NP 2189). A summary of patient demographic and clinical characteristics for all four cohorts are shown in Table 1.

**TABLE 1:** The demographic information for cohorts 1–4.

	*N*	Age, mean (SD)	Sex, %F	*APOE* ε4 carriership, %	MMSE	AD CSF biomarker characterization	AD biomarker method
**Cohort 1**							
Non‐AD	15	63.1 (5.2)	66.7%	26.7%	NA	0.093 (0.022)	CSF A β 42/40 > 0.065 (Lumipulse G1200)
AD	25	65.2 (3.4)	64%	72%	NA	0.045 (0.012)	CSF A β 42/40 > 0.065 (Lumipulse G1200)
**Cohort 2**							
MCI+	25	73.8 (6.2)	64%	13.3%	23.9 (3.9)	4,9 (5,1)	CSF Aβ42/p‐tau ratio < 10.25 (Lumipulse G1200)
MCI–	15	73.4 (4,6)	60%	56%	24.3 (4.3)	23,8 (7,1)	CSF Aβ42/p‐tau ratio > 10.25 (Lumipulse G1200)
**Cohort 3**							
AD^a^	30	77.2 (7.1)	50%	66.7%	21.4 (5.5)	1.02 (0.07)	CSF p‐tau/ Aβ42 ratio > 0.9
LB^b^	24^a^	69.3 (4.1)	41.6%	25%	21.6 (4.8)	0,74 (0.51)	CSF p‐tau/Aβ42 ratio < 0.9
**Cohort 4**							
GRN–	20	65.4 (5.2)	65%	NA	NA	NA	NA
GRN+	20^d^	63.1 (4.7)	65%	NA	NA	NA	NA

Abbreviations: Aβ, amyloid beta; AD, Alzheimer's disease; *APOE*, apolipoprotein E; CSF, cerebrospinal fluid; GRN, progranulin; LB, Lewy body; MCI, mild cognitive impairment; MMSE, Mini‐Mental State Examination; p‐tau, phosphorylated tau; SAA, seed amplification assay; SD, standard deviation.

^a^ Negative for SAA.

^b^ Positive for SAA.

^c^ Dementia with Lewy bodies, *n* = 22; Parkinson's disease, *n* = 2.

^d^ Agrammatic variant of primary progressive aphasia, *n* = 11; behavioral variant of primary progressive aphasia, *n* = 6; semantic variant of primary progressive aphasia, *n* = 1; pre‐symptomatic, *n* = 2.

### 2.2 NULISA analysis

NULISA assays were performed at Alamar Biosciences as described previously.^44^ Briefly, plasma, serum, and CSF samples stored at –80°C were thawed on ice and centrifuged at 10,000 × g for 10 minutes. Exactly 10 µL supernatant were plated in 96‐well plates and analyzed with Alamar's CNS Disease Panel targeting mostly neurodegenerative disease–related targets as well as inflammation and immune response–related cytokines and chemokines. A Hamilton‐based automation instrument was used to perform the NULISAseq workflow. Briefly, immunocomplexes were formed with DNA‐barcoded capture and detection antibodies, followed by a capturing and washing step of the immunocomplexes on paramagnetic oligo‐dT beads. The immunocomplexes were then released into a low‐salt buffer, which were captured and washed on streptavidin beads. Finally, the proximal ends of the DNA strands on each immunocomplex were ligated to generate a DNA reporter molecule containing both target‐specific and sample‐specific barcodes. DNA reporter molecules were pooled and amplified by polymerase chain reaction (PCR), purified, and sequenced on Illumina NextSeq 2000. Singleplex NULISA assays were performed by Alamar Bioscience on the Argo prototype instrument. For the p‐tau217 assay, 20 µL of plasma or CSF were used in a reaction volume of 100 µL containing capture and detection antibody cocktails in assay diluent buffer. The mixture was incubated at room temperature for 1 hour to allow the formation of immunocomplexes. These immunocomplexes then underwent two rounds of affinity bead purification and multiple washes, followed by ligation to form reporter molecules, which were then eluted. The final eluate was collected for quantification by quantitative PCR.

### 2.3 Data processing and normalization

For NULISAseq, sequencing data were processed using the NULISAseq algorithm (Alamar Biosciences). The sample‐ (SMI) and target‐specific (TMI) barcodes were quantified, and up to two mismatching bases or one indel and one mismatch were allowed. Intraplate normalization was performed by dividing the target counts for each sample well by that well's internal control counts. Interplate normalization was then performed using interplate control (IPC) normalization, wherein counts were divided by target‐specific medians of the three IPC wells on that plate. Data were then rescaled, add 1 and log2 transformed to obtain NULISA Protein Quantification (NPQ) units for downstream statistical analysis. For singleplex assays, the Cq readout from the calibrators and samples was transformed to a linear scale using the formula 2^(37−Cq). The linear transformed signal was then used in the 4‐parameter logistic (4‐PL) curve fitting for standard curve generation and sample analyte concentration calculation.

### 2.4 Statistical analysis

All statistical analyses and plots were performed on R statistical software version 4.4.0. For descriptive statistics chi‐square tests compared categorical variables while one‐way analysis of variance compared continuous variables between groups (AD vs. non‐AD [cohort 1]; MCI+ vs. MCI—[cohort 2]; LB vs. AD [cohort 3]; GRN+ vs. GRN– [cohort 4]). Linear Models for Microarray and RNA‐Seq Data (LIMMA models) evaluated the differential protein expression between designated groups, as described above, for which the target proteins were set as the dependent variable and the group as the predictor variable. Volcano plots report by color schemes the proteins that were nominally significant as well as the proteins that remained significant after false discovery rate (FDR) correction for multiple comparisons. Spearman rank tested for correlations between target proteins across fluid matrices (plasma vs. serum; plasma vs. CSF; serum vs. CSF) and confidence intervals (CI) at 95% were determined with 1000 bootstraps resampling. The area under the receiver operating characteristic curve (AUROC) assessed the biomarker accuracy to distinguish predefined statuses and 95% CI of sensitivities and specificities were also computed (Youden index).

## 3 RESULTS

This study included four pilot datasets totaling 190 participants (Table 1); 40 participants (mean [standard deviation (SD)] age, 63.8 [5.9] years; *n* [%] 17 females [42.5%]) biologically determined as AD or non‐AD by CSF biomarkers (cohort 1), from the Alzheimer's Association GBSC plasma phospho‐tau Round Robin study; 40 participants (mean [SD] age, 73.7 [5.9] years; *n* [%] 25 females [62.5%]) clinically determined identified as MCI with or without AD pathology determined by CSF Aβ42/p‐tau181 (cohort 2); 54 participants (mean [SD] age, 76.3 [7.8] years; *n* [%] 24 females [44.4%]) with or without LB pathology determined by SAA and AD pathology determined by CSF p‐tau181/Aβ42 (cohort 3), and 40 participants (mean [SD] age, 63.9 [6.9] years; *n* [%] 26 females [65.0%]) with (*n* = 20) or without a *GRN* mutation (cohort 4).

### 3.1 NULISAseq in biomarker‐defined AD compared to non‐AD

**FIGURE 1:**
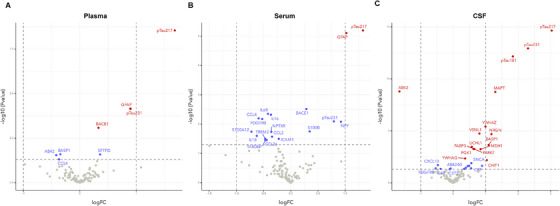
Differential protein changes between biologically determined AD compared to non‐AD (cohort 1) in plasma (A), serum (B), and CSF (C). AD, Alzheimer's disease; CSF, cerebrospinal fluid; FC, fold change.

In the plasma of biologically determined AD compared to non‐AD (cohort 1), we observed six differentially deregulated proteins (Figure 1A), of which four upregulated proteins in AD passed multiple testing correction (p‐tau217, logFC = 1.68, *P_adj_* < 0.001; GFAP, logFC = 0.90, *P_adj_* = 0.003; p‐tau231, logFC = 0.90, *P_adj_* = 0.003; BACE1, logFC = 0.33, *P_adj_* = 0.02; Figure S1 in supporting information). The boxplots of nominally significant proteins are shown in Figure S2 in supporting information. Interestingly, in the serum of the same patients, we observed 18 significantly changed proteins (Figure 1B) of which two upregulated proteins passed multiple testing correction (p‐tau217, logFC = 1.33, *P_adj_* < 0.001; GFAP, logFC = 1.02, *P_adj_* < 0.001; Figure S3 in supporting information). The boxplots of nominally significant proteins are shown in Figure S4 in supporting information. In the CSF of the same patients, 22 proteins significantly changed were observed (Figure 1C), of which 1 downregulated and 14 upregulated proteins passed multiple testing correction (Figure S5 in supporting information). The boxplots of nominally significant proteins are shown in Figure S6 in supporting information. The results for all proteins measured in the NULISAseq CNS panel for plasma, serum, and CSF are displayed in Tables S1–S3 in supporting information.

### 3.2 NULISAseq measurements of p‐tau217

In cohort 1, we also compared NULISAseq multiplex p‐tau217 measures to other high‐performing p‐tau217 singleplex immunoassays previously described in the literature (Figure S7 in supporting information). We found a high correlation between NULISAseq p‐tau217 levels and Janssen p‐tau217+ (*r* = 0.924, *P* < 0.001), ALZpath p‐tau217 (*r* = 0.924, *P* < 0.001), Lumipulse p‐tau217 (*r* = 0.936, *P* < 0.001) and mass spectrometry p‐tau217 measures (*r* = 0.925, *P* < 0.001) with differing fold‐changes but similar diagnostic accuracy. NULISAseq and NULISA singleplex measures of p‐tau217 strongly correlated (*r* = 0.906, *P* < 0.00). We observed a larger fold‐change for the NULISA singleplex compared to NULISAseq but this did not translate to statistically different diagnostic accuracy.

### 3.3 NULISAseq in patients with MCI due to AD (Aβ+) compared to MCI unlikely to be due to AD (Aβ–)

**FIGURE 2:**
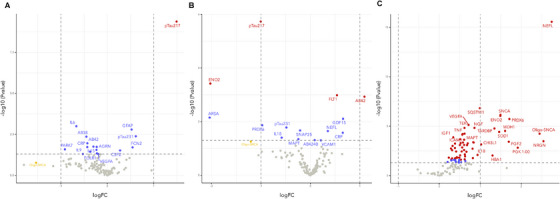
MCI due to AD patients and MCI unlikely due to AD (A), LB+ compared to LB– (B), and *GRN* mutation carriers compared to non‐carriers (C). AD, Alzheimer's disease; FC, fold change; *GRN*, progranulin; LB, Lewy body; MCI, mild cognitive impairment.

In the plasma of patients with MCI due to AD compared to MCI unlikely to be due to AD (cohort 2), we observed 17 differentially changed proteins (Figure 2A) but only the upregulation of p‐tau217 protein that passed multiple testing correction (p‐tau217, logFC = 1.49, *P_adj_* < 0.001); Figure S8 in supporting information). Of the nominally significant proteins (Figure S9 in supporting information), several cytokines and immune‐related proteins (interleukin [IL]‐6, logFC = –0.668, *P *= 0.001; tumor necrosis factor [TNF], logFC = –0.299, *P *= 0.017; C‐reactive protein [CRP], logFC = –0.45, *P =* 0.018; IL‐15, logFC = –0.23, *P *= 0.027; IL‐9, logFC = 0.54, *P* = 0.49), amyloid peptides (Aβ38, logFC = –0.45, *P* = 0.004; Aβ42, logFC = –0.43, *P* = 0.011) and proteins implicated in synucleinopathies (PARK7, logFC = –0.912, *P *= 0.027; FABP3, logFC = –0.370, *P* = 0.036) were downregulated. GFAP (logFC = 0.521, *P *= 0.002) and p‐tau231(logFC = 0.611, *P *= 0.004) were upregulated. In addition, despite not being statistically significant, oligomeric α‐synuclein (Oligo‐SNCA) had a larger fold‐change than p‐tau217 (Oligo‐SNCA, logFC = –1.54, *P_adj_* > 0.05; Figure 2A). The results for all proteins measured in the multiplex for plasma are displayed in Table S4 in supporting information.

### 3.4 NULISAseq in LB SAA+ patients compared to AD SAA– patients

In the plasma of symptomatic patients with and without αSyn pathology, determine by CSF SAA, we observed 15 differentially changed proteins (Figure 2B), of which 2 proteins were upregulated (Aβ42, logFC = 1.09, *P_adj _* = 0.036; vascular endothelial growth factor receptor [FLT1], logFC = 0.537, *P_adj_* = 0.036) and 2 were downregulated (p‐tau217, logFC = 1.01, *P_adj _* < 0.001; neuron specific enolase function [ENO2], logFC = –2.02, *P_adj_* = 0.023; Figure S10 in supporting information) passed multiple testing correction. The boxplots of nominally significant proteins are shown in Figure S11 in supporting information. We also selectively examined proteins in the CNS panel which were related to αSyn (αSyn, Oligo‐SNCA, phosphorylated αSyn‐129; Figure S12 in supporting information). Interestingly, we found a non‐significant change of Oligo‐SNCA but with a similar fold‐change to p‐tau217 (logFC = –1.05, *P *> 0.05). The results for all proteins measured in the multiplex for plasma are displayed in Table S5 in supporting information.

### 3.5 NULISAseq in *GRN* mutation carriers compared to non‐carriers

In the plasma of *GRN* mutation carriers we observed 65 differentially upregulated proteins (Figure 2C), of which 55 proteins passed multiple testing correction compared to non‐mutation carriers (Table S6 in supporting information). The most significantly changed protein was NfL (logFC = 2.74, *P_adj _* < 0.001; Figure S13 in supporting information). Notably, neurogranin (logFC = 2.54, *P_adj _* < 0.001; Figure S14), Oligo‐SNCA (logFC = 2.46, *P_adj _* < 0.001; Figure S14) demonstrated similar fold‐changes to NfL. Further, superoxide dismutase 1 (logFC = 2.46, *P_adj _* < 0.001; Figure S14 in supporting information) and sequestosome 1 (logFC = 2.46, *P_adj _* < 0.001; Figure S14) and TDP‐43 (logFC = 2.46, P*_adj _* < 0.001; Figure S14) all showed significantly different changes between groups.

### 3.6 Detectability differences between plasma and serum

As alluded to above, commonalities, but many more differences, are observed between plasma and serum when comparing diagnostic groups. To illustrate this further, we correlated the same proteins detected by NULISA between plasma and serum (Figure S15; Table S7 in supporting information). Interestingly, 30.9% of proteins were not significantly correlated between plasma and serum. Of note, p‐tau181 (*r* = 0.019), p‐tau231 (r = 0.187), Aβ38 (*r* = 0.172), Aβ42 (*r* = 0.101), t‐tau (*r* = 0.161), TDP‐43 (*r* = –0.058), pTDP‐43 (*r* = 0.005), all had poor and non‐significant relationships between plasma and serum. Within the significantly correlated proteins, a wide range of correlation coefficients were overserved (*r* = 0.33‐0.971). Neurofilament heavy chain (*r* = 0.971; *P* = 2.4^−25^) and NfL (*r* = 0.93; *P* = 4.1^−17^) were among the most highly correlated proteins. P‐tau217 (*r* = 0.662; *P* = 3.6^−6^) and GFAP (*r* = 0.887; *P* = 2.7^−14^) had significant correlations between plasma and serum. Next, we compared the correlations among plasma, serum, and CSF. We observed that 25.6% of proteins were significantly correlated (*P* < 0.05) between plasma and CSF (Figure S16; Table S8 in supporting information). The most significantly correlated proteins included CRP (*r* = –898; *P* = 5.2^−13^), platelet‐derived growth factor receptor beta (PDGFRβ; *r* = 0.728; *P* = 9.9^−8^), p‐tau217 (*r* = 0.721; *P* = 1.5^−7^), and NfL (*r* = 0.701; *P* = 4.8^−7^). In serum (Figure S17; Table S9 in supporting information), only 16.7% of proteins were significantly correlated (*P* < 0.05) between serum and CSF. Like plasma, CRP (*r* = –887; *P* = 2.34^−14^), PDGFRβ (*r* = 0.634; *P* = 1.1^−6^), and NfL (*r* = 0.681; *P* = 1.3^−6^) were among the other tightly associated.

We also compared the relative abundance of proteins in plasma and serum (Figure S18 in supporting information) by creating a ratio of the NPQ values. The majority of proteins’ mean plasma‐to‐serum NPQ ratio were ≈ 1, meaning little difference in the abundance between the matrices. However, several proteins had a higher abundance in plasma (e.g., Oligo‐SNCA, Neurogranin, enolase 2) whereas S100b, GFAP, TNF, and IL‐6 has a higher abundance in serum.

## 4 DISCUSSION

The findings from this series of pilot studies highlight the potential of a new generation of multi‐protein measurement platforms in enhancing the detection and characterization of biomarkers for neurodegenerative diseases, particularly AD. While mass spectrometry analyses in blood remain the only truly unbiased discovery approach, they are hampered by the presence of highly abundant proteins. In contrast, new high‐plex antibody or aptamer technologies with attomolar sensitivity (e.g., NULISA) demonstrate a novel approach by combining robust single analytes with known utility (e.g., p‐tau217 and NfL) with a multi‐analyte screening capacity in low sample volume (< 25 µL).

In AD‐related cohorts, this study confirmed the utility of p‐tau217 as the leading biomarker in both plasma and serum. The consistent upregulation of p‐tau217 across different biological matrices (plasma, serum, and CSF), the correlation within such matrices, and with the NULISA p‐tau217 measures highly correlated with established single‐plex immunoassays and mass spectrometry measures, underscores the reliability of the biomarker and this novel multiplex measurement format. In concordance with previous work, p‐tau231 and GFAP were seen as ancillary biomarkers in AD. Fundamentally, the NULISA platform also revealed a contingent of biomarkers differentially expressing proteins in AD and MCI, which is more representative of the complexity of the disease and the potential for a multi‐marker approach to improve underlying pathophysiology of the disease. In participants of biologically determined AD compared to non‐AD we measured the multiplex panel in plasma, serum, and CSF. It is generally considered that plasma is the preferred matrix for AD blood biomarkers,^45^ with lower concentrations of key biomarkers (e.g., p‐tau) in serum despite good assocations,^46^ and in the case of Aβ42, weak correlations between the matrices.^46^ Comparing plasma and serum, we reveal differences in significant biomarker changes between AD and non‐AD but also some commonality, for example, p‐tau217 and GFAP. Intriguingly, we show that approximately one third of analyzed proteins were not significantly correlated between plasma and serum (correlation coefficient < 0.3) and this illustrates the importance of considering both plasma and serum as complementary biofluids, not interchangeable biofluids, for a thorough novel biomarker discovery in blood.

The study also explored biomarker profiles in other neurodegenerative conditions in an endophenotype approach, such as LB disease and *GRN* mutation carriers, one of the most common forms of genetic FTD. In LB pathology (SAA+ but Aβ–) compared to AD (SAA– but Aβ+), we confirmed the importance of p‐tau217 but also Aβ42 as discriminatory biomarkers between AD and non‐AD pathologies. However, significant changes in proteins vascular endothelial growth factor receptor, enolase 2, and GDF15 were also observed. A non‐significant but noticeable change in Oligo‐SNCA as a promising advancement for LB‐related biomarkers in plasma was also seen. Changes in Oligo‐SNCA were also observed in MCI– patients compared to MCI+ patients as well as *GRN* mutation carriers. The *GRN* mutation carriers pilot study observed a remarkable number of upregulation proteins, including NfL, neurogranin, and TDP‐43. The significant fold changes in these proteins indicate their potential utility in identifying and monitoring *GRN*‐related pathologies and possible translatable blood biomarkers for sporadic forms of FTD.

Despite these promising results, the study acknowledges several limitations. The group comparisons included in this pilot study are not sufficiently powered to draw definitive conclusions regarding the novel biomarkers identified or the proteins that did not reach statistical significance but exhibited change, which were numerous. This limitation underscores the necessity for larger, more robust cohorts to validate these preliminary findings. Yet, the independent validation of p‐tau217, GFAP, and NfL in AD and *GRN* mutation carriers, which were unbiasedly ranked the highest, significantly enhances the credibility of these findings.

In conclusion, this study presents a promising new direction for clinical proteomics that use CNS‐dedicated multiplex panels which incorporate robust known targets but simultaneously allow for novel discovery. Such advances will offer the blood‐based diagnostic and prognostic tools for unmet needs within neurodegenerative disorders.

## CONFLICT OF INTEREST STATEMENT

HZ has served on scientific advisory boards and/or as a consultant for Abbvie, Acumen, Alector, Alzinova, ALZPath, Amylyx, Annexon, Apellis, Artery Therapeutics, AZTherapies, Cognito Therapeutics, CogRx, Denali, Eisai, LabCorp, Merry Life, Nervgen, Novo Nordisk, Optoceutics, Passage Bio, Pinteon Therapeutics, Prothena, Red Abbey Labs, reMYND, Roche, Samumed, Siemens Healthineers, Triplet Therapeutics, and Wave, has given lectures in symposia sponsored by Alzecure, Biogen, Cellectricon, Fujirebio, Lilly, Novo Nordisk, and Roche, and is a co‐founder of Brain Biomarker Solutions in Gothenburg AB (BBS), which is a part of the GU Ventures Incubator Program (outside submitted work). BB has served on scientific advisory boards for Alector, Alexion, AviadoBio, Denali, Lilly/Prevail, UCB, and Wave. APi received consultancy/speaker fees from Abbvie, Bial, Lundbeck, Roche, and Zambon pharmaceuticals. APa received grant support from Ministry of Health (MINSAL) and Ministry of Education, Research and University (MIUR), from CARIPLO Foundation; personal compensation as a consultant/scientific advisory board member for Biogen, Lundbeck, Roche, Nutricia, General Healthcare (GE). MS‐C has given lectures in symposia sponsored by Almirall, Eli Lilly, Novo Nordisk, Roche Diagnostics, and Roche Farma; received consultancy fees (paid to the institution) from Roche Diagnostics; and served on advisory boards of Eli Lilly, Grifols, and Roche Diagnostics. He was granted a project and is a site investigator of a clinical trial (funded to the institution) by Roche Diagnostics. In‐kind support for research (to the institution) was received from ADx Neurosciences, Alamar Biosciences, Avid Radiopharmaceuticals, Eli Lilly, Fujirebio, Janssen Research & Development, and Roche Diagnostics. All other authors do not report any conflicts of interest. Author disclosures are available in the supporting information.

## CONSENT STATEMENT

All human subjects provided written or verbal informed consent. The study was approved by the local ethics committee from each participating site.

## Supporting information



Supporting Information

Supporting Information
