# Supplementary material for: Biomarker discovery in Alzheimer's and neurodegenerative diseases using Nucleic Acid Linked Immuno‐Sandwich Assay
Source: Alzheimers Dement. 2025 May 22;21(5):e14621. doi: 10.1002/alz.14621 (PMC12096316; doi:10.1002/alz.14621)
Supplement: Supplementary file 1 — Supporting Information [file ALZ-21-e14621-s001.docx]

**Biomarker Discovery in Alzheimer's and Neurodegenerative Diseases using Nucleic Acid-Linked Immuno-Sandwich Assay**

**Authors**

Nicholas J. Ashton^1,2,3*^, Andrea L. Benedet^1*^, Guglielmo Di Molfetta^1^, Ilaria Pola^1^, Federica Anastasi^4,5^, Aida Fernández-Lebrero^4,5,6,7^, Albert Puig-Pijoan^4,6,8^, Ashvini Keshavan^9^, Jonathan Schott^9,10^, Kubra Tan^1^, Joel Simrén^1^, Bárbara Fernandes Gomes^1^, Laia Montoliu-Gaya^1^, Richard Isaacson^11,12^, Matilde Bongianni^13^, Chiara Tolassi^14^, Valentina Cantoni^15^, Antonella Alberici^15^, Alessandro Padovani^15,16,17,18^, Gianluigi Zanusso^19^, Andrea Pilotto^15,16,17,18^, Barbara Borroni^15,16^, Marc Suárez-Calvet^4,5,6,20^, Kaj Blennow^1,21,22,23^, Henrik Zetterberg^1,9,10,21,24,25^

**Affiliations:**

^1^ Department of Psychiatry and Neurochemistry, Institute of Neuroscience & Physiology, the Sahlgrenska Academy at the University of Gothenburg, Wallinsgatan 6, Mölndal S-43141, Sweden; ^2^ Banner Alzheimer's Institute and University of Arizona, Phoenix, 901 E Willetta St AZ 85006, USA; ^3^ Banner Sun Health Research Institute, Sun City, 10515 W Santa Fe Drive, AZ 85351, USA; ^4^ Hospital del Mar Research Institute, C/ del Dr. Aiguader, 88, Ciutat Vella, 08003 Barcelona, Spain; ^5^ Barcelonaβeta Brain Research Center (BBRC), Pasqual Maragall Foundation, C/ de Wellington, 30, Sant Martí, 08005 Barcelona, Spain; ^6^ Servei de Neurologia, Hospital del Mar, C/ del Dr. Aiguader, 88, Ciutat Vella, 08003 Barcelona, Spain;^7^ Department of Medicine and Life Sciencesces, Universitat Pompeu Fabra,  C/ del Dr. Aiguader, 88, Ciutat Vella, 08003 Barcelona, Spain; ^8^ Department of Medicine, Universitat Autònoma de Barcelona, Gran Via de Les Corts Catalanes, Barcleona, Spain; ^9^ Dementia Research Centre, UCL Queen Square Institute of Neurology, University College London, Maple House, Tottenham Ct Rd, London W1T 7NF, UK; ^10^ UK Dementia Research Institute at UCL, Maple House, Tottenham Ct Rd, London W1T 7NF, UK; ^11^ Department of Neurology, Weill Cornell Medicine and New York - Presbyterian, NY 10022, New York, USA; ^12^ Department of Neurology, Florida Atlantic University, Charles E. Schmidt College of Medicine, Boca Raton, FL 33431, Florida, USA; ^13^ Department of Neurosciences, Biomedicine, and Movement Sciences, Policlinico G. B. Rossi, University of Verona, 37134, Verona, Italy; ^14^ Clinical Investigation in Laboratory, Maggiore Hospital ASST-Crema, Crema, Italy; ^15^ Department of Clinical and Experimental Sciences, Neurology Unit, University of Brescia, 25123 Brescia, Italy; ^16^ Department of Continuity of Care and Frailty, Azienda Socio Sanitaria Territoriale (ASST) Spedali Civili, 25123 Brescia, Italy; ^17^ Laboratory of Digital Neurology and Biosensors, University of Brescia, 25123 Brescia, Italy; ^18^ Brain Health Center, University of Brescia, via Fratelli Ugoni, 2, 25126 Brescia, Italy; ^19^ Department of Neuroscience, Biomedicine and Movement Sciences, University of Verona, 37134 Verona, Italy; ^20^ Centro de Investigación Biomédica en Red de Fragilidad y Envejecimiento Saludable (CIBERFES), Pabellón 11, 28029 Madrid, Spain; ^21^ Clinical Neurochemistry Laboratory, Sahlgrenska University Hospital, Mölndal, Wallinsgatan 6, Mölndal S-43141, Sweden; ^22^ Paris Brain Institute, ICM, Pitié-Salpêtrière Hospital, Sorbonne University, Hôpital Pitié, 47 Bd de l'Hôpital, 75013 Paris, France; ^23^ Neurodegenerative Disorder Research Center, Division of Life Sciences and Medicine, and Department of Neurology, Institute on Aging and Brain Disorders, University of Science and Technology of China and First Affiliated Hospital of USTC, 230027 Hefei, PR China; ^24^ Hong Kong Center for Neurodegenerative Diseases, Clear Water Bay, 17 Science Park W Ave, Science Park, Hong Kong, China; ^25^ Wisconsin Alzheimer's Disease Research Center, University of Wisconsin School of Medicine and Public Health, University of Wisconsin-Madison, Madison, WI, USA

**Table of contents**

**Supplementary tables**

**Table S1**- Comparison of plasma protein expression between AD and non-AD (first 50 results by *P* value).

**Table S2**- Comparison of serum protein expression between AD and non-AD (first 50 results by *P* value).

**Table S3**- Comparison of CSF protein expression between AD and non-AD (first 50 results by *P* value).

**Table S4**- Comparison of plasma protein expression between MCI+ and MCI- (first 50 results by *P* value).

**Table S5**- Comparison of plasma protein expression between LB+ and AD (first 50 results by *P* value).

**Table S6**- Comparison of plasma protein expression between *GRN*+ and *GRN*- (first 50 results by *P* value).

**Table S7**- Correlation of the same protein targets in plasma and serum (ranked by *P* value)

**Table S8**- Correlation of the same protein targets in plasma and CSF (ranked by *P* value)

**Table S9**- Correlation of the same protein targets in plasma and serum (ranked by *P* value)

**Supplementary figures**

**Figure S1.** Plasma proteins passing multiple testing correction in biologically determined AD compared to non-AD (cohort 1).

**Figure S2.** Nominally significant plasma proteins in biologically determined AD compared to non-AD (cohort 1).

**Figure S3.** Serum proteins passing multiple testing correction in biologically determined AD compared to non-AD (cohort 1).

**Figure S4.** Nominally significant serum proteins in biologically determined AD compared to non-AD (cohort 1).

**Figure S5.** CSF proteins passing multiple testing correction in biologically determined AD compared to non-AD (cohort 1).

**Figure S6**. Nominally significant CSF proteins in biologically determined AD compared to non-AD (cohort 1).

**Figure S7**. Comparison between NULISAseq plasma pTau217 and other assays.

**Figure S8.** Plasma proteins passing multiple testing correction in MCI Aβ+ compared to MCI Aβ-.

**Figure S9.** Nominally significant plasma proteins in MCI Aβ+ compared to MCI Aβ-.

**Figure S10.** Plasma proteins passing multiple testing correction in LB+ compared to AD.

**Figure S11.** Nominally significant plasma proteins in LB+ compared to AD.

**Figure S12.** Plasma proteins of interest compared between LB+ and AD.

**Figure S13.** Plasma proteins passing multiple testing correction in *GRN*+ compared to *GRN*-.

**Figure S14.** Plasma proteins of interest compared between *GRN*+ and *GRN*-.

**Figure S15** - Correlation of the same protein targets in plasma and serum (ranked Correlation coefficient)

**Figure S16** - Correlation of the same protein targets in plasma and serum (ranked Correlation coefficient)

**Figure S17** - Correlation of the same protein targets in plasma and serum (ranked Correlation coefficient)

**Figure S18** – The NPQ ratio between plasma and serum to compare abundance

**Table S1**- Comparison of plasma protein expression between AD and non-AD in cohort 1 (first 50 results by *P* value).

| ID | UniProtID | logFC | *t* | *B* | *P* value | Adj. *P* value |
| --- | --- | --- | --- | --- | --- | --- |
| pTau217 | P10636 | 1.68243151 | 7.65514351 | 11.1397931 | 2.84E-09 | 3.36E-07 |
| GFAP | P14136 | 0.89576973 | 4.45761813 | 1.28854857 | 6.87E-05 | 0.00285764 |
| pTau231 | P10636 | 0.89777693 | 4.43959288 | 1.22027634 | 7.27E-05 | 0.00285764 |
| BACE1 | P56817 | 0.33070934 | 3.62299917 | -1.0898619 | 0.00083191 | 0.02454131 |
| BASP1 | P80723 | -0.3447705 | -2.3255401 | -4.2620102 | 0.02536532 | 0.48067366 |
| SFTPD | P35247 | 0.36021297 | 2.31472682 | -4.2823952 | 0.02601139 | 0.48067366 |
| AB42 | P05067 | -0.4179194 | -2.2749878 | -4.3340006 | 0.02851454 | 0.48067366 |
| CCL4 | P13236 | -0.3689988 | -2.0317656 | -4.8106376 | 0.04906712 | 0.72374009 |
| POSTN | Q15063 | 0.30808589 | 1.92309067 | -5.0389012 | 0.06183166 | 0.81068172 |
| MME | P08473 | -0.97324 | -1.7177449 | -5.395637 | 0.09381732 | 0.9966394 |
| pTau181 | P10636 | 0.28406777 | 1.65009493 | -5.4909659 | 0.1069987 | 0.9966394 |
| PDGFRB | P09619 | -0.314521 | -1.5969889 | -5.5659395 | 0.11838344 | 0.9966394 |
| CRP | P02741 | -0.7070814 | -1.5727272 | -5.6242237 | 0.12390371 | 0.9966394 |
| TREM2 | Q9NZC2 | -0.3625838 | -1.5623343 | -5.6329837 | 0.12633144 | 0.9966394 |
| IL6R | P08887 | -0.1878761 | -1.532207 | -5.6745903 | 0.13356794 | 0.9966394 |
| NEFH | P12036 | 1.08588827 | 1.50880157 | -5.7187454 | 0.1394519 | 0.9966394 |
| PGF | P49763 | 0.17847253 | 1.4742682 | -5.7773369 | 0.14846031 | 0.9966394 |
| TNF | P01375 | -0.1550728 | -1.40046 | -5.8432747 | 0.16930526 | 0.9966394 |
| SNAP25 | P60880 | 0.17025212 | 1.37955187 | -5.8890562 | 0.17560724 | 0.9966394 |
| PTN | P21246 | 0.1753795 | 1.36611722 | -5.9014702 | 0.17975159 | 0.9966394 |
| IL16 | Q14005 | -0.2507351 | -1.3296976 | -5.9492979 | 0.19138434 | 0.9966394 |
| SQSTM1 | Q13501 | -0.4039895 | -1.3361665 | -5.9530043 | 0.18928048 | 0.9966394 |
| PDLIM5 | Q96HC4 | 0.60047048 | 1.27653618 | -6.0066181 | 0.20935544 | 0.9966394 |
| S100B | P04271 | 0.23386383 | 1.23244941 | -6.0769993 | 0.22519891 | 0.9966394 |
| FABP3 | P05413 | -0.1587092 | -1.1782927 | -6.1240493 | 0.24584241 | 0.9966394 |
| AGRN | O00468 | -0.1330203 | -1.1748813 | -6.1292233 | 0.247187 | 0.9966394 |
| CD40LG | P29965 | 0.4134112 | 1.16628366 | -6.1490518 | 0.25062017 | 0.9966394 |
| SFRP1 | Q8N474 | 0.2690654 | 1.13640349 | -6.1939293 | 0.2627595 | 0.9966394 |
| REST | Q13127 | -0.2407614 | -1.12284 | -6.1978163 | 0.268407 | 0.9966394 |
| NPY | P01303 | 0.14256686 | 1.070425 | -6.2258875 | 0.29102774 | 0.9966394 |
| ICAM1 | P05362 | -0.1249944 | -1.0862994 | -6.252303 | 0.28403515 | 0.9966394 |
| CCL3 | P10147 | -0.1820432 | -1.0419187 | -6.2733379 | 0.30389962 | 0.9966394 |
| VSNL1 | P62760 | 0.17026804 | 1.04901982 | -6.2907177 | 0.30066089 | 0.9966394 |
| IL5 | P05113 | -0.2649183 | -1.023311 | -6.2997963 | 0.31250022 | 0.9966394 |
| PARK7 | Q99497 | -0.5747687 | -1.0257999 | -6.3127942 | 0.31134025 | 0.9966394 |
| NRGN | Q92686 | 0.28468994 | 0.99212968 | -6.3533962 | 0.32728278 | 0.9966394 |
| CCL11 | P51671 | 0.10093057 | 0.93520249 | -6.3642812 | 0.35545592 | 0.9966394 |
| SLIT2 | O94813 | 0.08251242 | 0.91919466 | -6.3659981 | 0.36366103 | 0.9966394 |
| FOLR1 | P15328 | -0.1789906 | -0.9791799 | -6.3675004 | 0.33355858 | 0.9966394 |
| MAPT | P10636 | 0.18919334 | 0.91598973 | -6.41209 | 0.36533173 | 0.9966394 |
| IFNG | P01579 | 0.32568519 | 0.90802335 | -6.4296075 | 0.3694726 | 0.9966394 |
| CD63 | P08962 | -0.1995834 | -0.8861406 | -6.4507284 | 0.38100239 | 0.9966394 |
| CCL2 | P13500 | -0.1743654 | -0.842569 | -6.4518199 | 0.40463462 | 0.9966394 |
| S100A12 | P80511 | -0.2617003 | -0.8388548 | -6.4617291 | 0.40669045 | 0.9966394 |
| VEGFD | O43915 | 0.06506711 | 0.8190074 | -6.4626141 | 0.41777376 | 0.9966394 |
| CRH | P06850 | 0.31274531 | 0.86616937 | -6.4642278 | 0.39172312 | 0.9966394 |
| CXCL8 | P10145 | 0.23579937 | 0.87022533 | -6.4709858 | 0.38953058 | 0.9966394 |
| AB4240 | P123 | -0.0301305 | -0.8150959 | -6.4851249 | 0.4199821 | 0.9966394 |
| TEK | Q02763 | -0.0848925 | -0.8255047 | -6.4935444 | 0.41412131 | 0.9966394 |
| NPTXR | O95502 | -0.1070721 | -0.6392729 | -6.5235252 | 0.52640264 | 0.9966394 |

**Table S2**- Comparison of serum protein expression between AD and non-AD in cohort 1 (first 50 results by *P* value).

| ID | UniProtID | logFC | *t* | *B* | *P* value | Adj. *P* value |
| --- | --- | --- | --- | --- | --- | --- |
| pTau217 | P10636 | 1.32674659 | 5.19653673 | 3.79389929 | 6.36E-06 | 0.00048141 |
| GFAP | P14136 | 1.02026848 | 5.13163339 | 3.60170785 | 7.83E-06 | 0.00048141 |
| BACE1 | P56817 | 0.28574015 | 3.15170457 | -1.9878357 | 0.00307118 | 0.11389169 |
| IL6R | P08887 | -0.4195099 | -3.0131205 | -2.3417834 | 0.0044693 | 0.11389169 |
| IL16 | Q14005 | -0.364633 | -2.9857114 | -2.3895248 | 0.00480891 | 0.11389169 |
| CCL4 | P13236 | -0.5916711 | -2.8787184 | -2.6619046 | 0.00638897 | 0.11389169 |
| PDGFRB | P09619 | -0.5246538 | -2.8600409 | -2.6997975 | 0.00670847 | 0.11389169 |
| pTau231 | P10636 | 0.79148932 | 2.78766205 | -2.8627821 | 0.00809263 | 0.11389169 |
| NPY | P01303 | 0.921005 | 2.77625712 | -2.8902198 | 0.00833354 | 0.11389169 |
| S100B | P04271 | 0.34699461 | 2.47081186 | -3.5910049 | 0.01783674 | 0.18934934 |
| S100A12 | P80511 | -0.7226721 | -2.463605 | -3.5937374 | 0.0181652 | 0.18934934 |
| NPTXR | O95502 | -0.3577982 | -2.4562854 | -3.5943815 | 0.01847311 | 0.18934934 |
| IL18 | Q14116 | -0.6250294 | -2.3252012 | -3.8899468 | 0.02523404 | 0.23642842 |
| CCL3 | P10147 | -0.3440906 | -2.2972943 | -3.8986543 | 0.02691055 | 0.23642842 |
| TREM2 | Q9NZC2 | -0.4694577 | -2.2248034 | -4.0827687 | 0.03181648 | 0.24058007 |
| ICAM1 | P05362 | -0.2213658 | -2.22401 | -4.0877187 | 0.03185468 | 0.24058007 |
| CCL26 | Q9Y258 | -0.4434529 | -2.1801455 | -4.14573 | 0.03520684 | 0.24058007 |
| TARDBP | Q13148 | -0.4659201 | -2.1826069 | -4.1663601 | 0.03501195 | 0.24058007 |
| PGK1 | P00558 | -1.4947583 | -1.9740672 | -4.5730382 | 0.0553273 | 0.35817146 |
| TNF | P01375 | -0.2278038 | -1.8658402 | -4.7242254 | 0.06940496 | 0.42684049 |
| ANXA5 | P08758 | -0.5828074 | -1.7943104 | -4.8549455 | 0.08034534 | 0.43534379 |
| CRP | P02741 | -0.7661674 | -1.785205 | -4.8983361 | 0.08183365 | 0.43534379 |
| SNAP25 | P60880 | 0.15965326 | 1.75311494 | -4.9301897 | 0.08723968 | 0.43534379 |
| FOLR1 | P15328 | -0.2203494 | -1.752323 | -4.9304537 | 0.08737745 | 0.43534379 |
| FLT1 | P17948 | -0.1936706 | -1.7459968 | -4.9530815 | 0.08848451 | 0.43534379 |
| AGRN | O00468 | -0.1505404 | -1.62458 | -5.1160214 | 0.1121029 | 0.52157158 |
| PARK7 | Q99497 | -1.1980283 | -1.6136331 | -5.1686457 | 0.11449132 | 0.52157158 |
| pTau181 | P10636 | 0.40431613 | 1.56703207 | -5.2066095 | 0.12500687 | 0.54913733 |
| MDH1 | P40925 | -0.2726312 | -1.5448869 | -5.2409569 | 0.13027112 | 0.55252922 |
| IGFBP7 | Q16270 | -0.1338146 | -1.4975087 | -5.3018649 | 0.14210705 | 0.58263892 |
| pTDP43 | Q13148 | -0.1701598 | -1.3515712 | -5.494824 | 0.18410286 | 0.68192639 |
| TREM1 | Q9NP99 | -0.2544852 | -1.3697211 | -5.5090145 | 0.17843569 | 0.68192639 |
| IL33 | O95760 | -0.1841315 | -1.3433091 | -5.5126081 | 0.18673986 | 0.68192639 |
| NEFH | P12036 | 0.92680886 | 1.3379212 | -5.5419284 | 0.18849998 | 0.68192639 |
| GDNF | P39905 | -0.9020513 | -1.3089697 | -5.5559348 | 0.1980356 | 0.69595367 |
| IFNG | P01579 | 0.43415611 | 1.26076883 | -5.6426443 | 0.2147169 | 0.6994581 |
| TEK | Q02763 | -0.1068355 | -1.2282692 | -5.6842049 | 0.22652036 | 0.6994581 |
| CCL11 | P51671 | 0.28095512 | 1.22447569 | -5.6875941 | 0.22795537 | 0.6994581 |
| NPTX2 | P47972 | -0.1246943 | -1.2127971 | -5.6888687 | 0.23231777 | 0.6994581 |
| REST | Q13127 | -0.1802953 | -1.1510215 | -5.702273 | 0.25655641 | 0.6994581 |
| IL17A | Q16552 | 0.37773415 | 1.16420408 | -5.7476082 | 0.25125738 | 0.6994581 |
| CSF2 | P04141 | -0.1847611 | -1.1505685 | -5.7504953 | 0.25675481 | 0.6994581 |
| CRH | P06850 | 0.33000947 | 1.15823198 | -5.7584048 | 0.2536571 | 0.6994581 |
| MME | P08473 | -0.7643801 | -1.1509714 | -5.7605019 | 0.25659679 | 0.6994581 |
| IL5 | P05113 | -0.2999525 | -1.1401226 | -5.771425 | 0.26103494 | 0.6994581 |
| CCL13 | Q99616 | -0.1849153 | -1.1116046 | -5.7834155 | 0.27295926 | 0.6994581 |
| FABP3 | P05413 | -0.1537871 | -1.1204858 | -5.7891593 | 0.26918574 | 0.6994581 |
| NPTX1 | Q15818 | 0.16739421 | 1.0847639 | -5.8006696 | 0.28452097 | 0.7142057 |
| FCN2 | Q15485 | -0.2096082 | -1.1190463 | -5.8141224 | 0.26981397 | 0.6994581 |
| YWHAZ | P63104 | -0.5228127 | -1.0688333 | -5.8573383 | 0.2915704 | 0.71726317 |

**Table S3**- Comparison of CSF protein expression between AD and non-AD in cohort 1 (first 50 results by *P* value).

| ID | UniProtID | logFC | *t* | *B* | *P* value | Adj. *P* value |
| --- | --- | --- | --- | --- | --- | --- |
| pTau217 | P10636 | 3.03435342 | 12.3977141 | 24.885293 | 2.24E-15 | 2.65E-13 |
| pTau231 | P10636 | 2.30350496 | 10.9288587 | 20.9288617 | 1.18E-13 | 6.95E-12 |
| pTau181 | P10636 | 1.83724058 | 10.3067148 | 19.1744638 | 6.86E-13 | 2.70E-11 |
| AB42 | P05067 | -1.6550807 | -7.7443077 | 11.4533453 | 1.61E-09 | 4.24E-08 |
| MAPT | P10636 | 1.2971381 | 7.71064743 | 11.3179016 | 1.79E-09 | 4.24E-08 |
| YWHAZ | P63104 | 0.99503472 | 5.34138503 | 3.69957368 | 3.83E-06 | 7.53E-05 |
| VSNL1 | P62760 | 0.808747 | 4.85582595 | 2.16998707 | 1.82E-05 | 0.00030023 |
| NRGN | Q92686 | 1.17781584 | 4.82110458 | 2.04641871 | 2.04E-05 | 0.00030023 |
| BASP1 | P80723 | 1.09029356 | 4.04684486 | -0.291277 | 0.00022721 | 0.00297902 |
| UCHL1 | P09936 | 0.57396341 | 3.91323201 | -0.6881887 | 0.00034008 | 0.00401293 |
| FABP3 | P05413 | 0.63090538 | 3.76334089 | -1.1092759 | 0.00053156 | 0.00485892 |
| PARK7 | Q99497 | 0.64804837 | 3.75051727 | -1.1320253 | 0.0005521 | 0.00485892 |
| PGK1 | P00558 | 0.6302407 | 3.74412603 | -1.1385224 | 0.00056262 | 0.00485892 |
| MDH1 | P40925 | 0.84165111 | 3.73587727 | -1.1943061 | 0.00057648 | 0.00485892 |
| YWHAG | P61981 | 0.3691298 | 3.01217229 | -3.1317874 | 0.00445331 | 0.03503269 |
| CHIT1 | Q13231 | 1.03557775 | 2.87084031 | -3.4383185 | 0.00647889 | 0.04778179 |
| SNCA | P37840 | 0.55942304 | 2.61155645 | -4.0977552 | 0.01257858 | 0.08731017 |
| CXCL10 | P02778 | -0.4721216 | -2.4324539 | -4.4822481 | 0.01950118 | 0.12784109 |
| TIMP3 | P35625 | 0.49211591 | 2.36201859 | -4.6177237 | 0.02306258 | 0.1358092 |
| CRP | P02741 | 0.88787209 | 2.36424195 | -4.6492648 | 0.02294175 | 0.1358092 |
| GOT1 | P17174 | 0.44454098 | 2.34212614 | -4.6903525 | 0.02416943 | 0.1358092 |
| SOD1 | P00441 | 0.3853024 | 2.1770923 | -5.0240625 | 0.03535228 | 0.1896168 |
| PDGFRB | P09619 | -0.5408532 | -2.1253699 | -5.1158573 | 0.03969783 | 0.19518102 |
| ENO2 | P09104 | 0.55052654 | 2.12769849 | -5.1348075 | 0.03949252 | 0.19518102 |
| AB4240 | P123 | -0.0800184 | -2.105217 | -5.1622907 | 0.04149016 | 0.19583357 |
| IL6R | P08887 | -0.3876549 | -2.0668403 | -5.232066 | 0.04517625 | 0.1986223 |
| BACE1 | P56817 | 0.33846505 | 2.06410754 | -5.2403252 | 0.04544748 | 0.1986223 |
| CST3 | P01034 | -0.1081603 | -2.0306521 | -5.2982219 | 0.04885947 | 0.20590775 |
| CALB2 | P22676 | 0.26912895 | 1.98093114 | -5.3369736 | 0.05441119 | 0.21387404 |
| NEFH | P12036 | -0.4858045 | -1.9757349 | -5.3863798 | 0.05501881 | 0.21387404 |
| SLIT2 | O94813 | 0.26871864 | 1.96588054 | -5.4251861 | 0.05618725 | 0.21387404 |
| PRDX6 | P30041 | 0.26321344 | 1.90943435 | -5.5296897 | 0.06330101 | 0.2323845 |
| RUVBL2 | Q9Y230 | -0.2098911 | -1.8967743 | -5.5514946 | 0.06498889 | 0.2323845 |
| IL5 | P05113 | 0.16193799 | 1.80510129 | -5.6790883 | 0.07846164 | 0.26300392 |
| IFNG | P01579 | -0.482184 | -1.7774806 | -5.7641015 | 0.08299221 | 0.26300392 |
| CD63 | P08962 | 0.18301776 | 1.74754827 | -5.7708887 | 0.08808531 | 0.26300392 |
| SQSTM1 | Q13501 | 0.25517284 | 1.74165137 | -5.7880363 | 0.08915387 | 0.26300392 |
| IL6 | P05231 | -0.2592682 | -1.7465113 | -5.7921442 | 0.08829631 | 0.26300392 |
| SFRP1 | Q8N474 | 0.77081017 | 1.75491083 | -5.7966306 | 0.08683043 | 0.26300392 |
| SAA1 | P0DJI8 | -0.6217746 | -1.7435629 | -5.8232539 | 0.08881575 | 0.26300392 |
| IL10 | P22301 | -0.5620414 | -1.7284921 | -5.8418868 | 0.09151089 | 0.2633728 |
| TARDBP | Q13148 | -0.1532707 | -1.6463422 | -5.9639973 | 0.10739597 | 0.30173154 |
| CD40LG | P29965 | -0.4740962 | -1.5739595 | -6.0426739 | 0.12326997 | 0.33015738 |
| IL4 | P05112 | -0.3615786 | -1.5815235 | -6.0566577 | 0.12153024 | 0.33015738 |
| CHI3L1 | P36222 | 0.26611784 | 1.56265543 | -6.1053208 | 0.12590748 | 0.33015738 |
| NEFL | P07196 | 0.31198139 | 1.51139369 | -6.133557 | 0.13844504 | 0.35514162 |
| CCL3 | P10147 | 0.22351322 | 1.45574669 | -6.2381489 | 0.153164 | 0.37816547 |
| PSEN1 | P49768 | 0.20668048 | 1.4469901 | -6.2615165 | 0.15558877 | 0.37816547 |
| IL7 | P13232 | 0.32075477 | 1.43173785 | -6.2940825 | 0.15988431 | 0.37816547 |
| PGF | P49763 | -0.2936371 | -1.4304907 | -6.3050761 | 0.16023961 | 0.37816547 |

**Table S4**- Comparison of plasma protein expression between MCI+ and MCI- (first 50 results by *P* value).

| ID | UniProtID | logFC | *t* | *B* | *P v*alue | Adj. *P* value |
| --- | --- | --- | --- | --- | --- | --- |
| pTau217 | P10636 | 1.49084953 | 8.24616777 | 12.9955608 | 4.17E-10 | 4.92E-08 |
| IL6 | P05231 | -0.6678983 | -3.5536975 | -1.3321434 | 0.00100563 | 0.05933243 |
| GFAP | P14136 | 0.52342787 | 3.38975674 | -1.8020134 | 0.00160291 | 0.06304763 |
| pTau231 | P10636 | 0.61359182 | 3.04425922 | -2.7026424 | 0.00414636 | 0.10265746 |
| AB38 | P05067 | -0.4520076 | -3.0263695 | -2.7580663 | 0.00434989 | 0.10265746 |
| AB42 | P05067 | -0.4286731 | -2.6694332 | -3.6135894 | 0.01098583 | 0.21605459 |
| TNF | P01375 | -0.2995502 | -2.4779621 | -4.0332394 | 0.01760959 | 0.23126887 |
| AGRN | O00468 | -0.2303654 | -2.4603674 | -4.0723138 | 0.01837253 | 0.23126887 |
| CRP | P02741 | -0.4318147 | -2.4508565 | -4.068974 | 0.01879733 | 0.23126887 |
| FCN2 | Q15485 | 0.54274533 | 2.4334265 | -4.0962155 | 0.01959906 | 0.23126887 |
| PARK7 | Q99497 | -0.9142411 | -2.3186486 | -4.3672355 | 0.02570627 | 0.27343754 |
| IL15 | P40933 | -0.2344156 | -2.2846528 | -4.4389861 | 0.02780721 | 0.27343754 |
| CSF2 | P04141 | 0.27490441 | 2.24755459 | -4.4375113 | 0.03028399 | 0.27488547 |
| FABP3 | P05413 | -0.3703325 | -2.1719325 | -4.6360105 | 0.03594988 | 0.30300614 |
| FOLR1 | P15328 | -0.2234717 | -2.064581 | -4.8522589 | 0.04559938 | 0.34050389 |
| IL9 | P15248 | -0.5269676 | -2.0236433 | -4.9364632 | 0.04983745 | 0.34050389 |
| VEGFA | P15692 | -0.1936505 | -2.0221626 | -4.9539012 | 0.04999697 | 0.34050389 |
| IGFBP7 | Q16270 | -0.1413628 | -2.0044375 | -4.9452843 | 0.05194127 | 0.34050389 |
| CCL2 | P13500 | -0.3978338 | -1.9740367 | -5.0459772 | 0.05542907 | 0.34424372 |
| IL33 | O95760 | -0.2910771 | -1.9315346 | -5.1056667 | 0.06064387 | 0.35779883 |
| CX3CL1 | P78423 | -0.1719156 | -1.8547655 | -5.2196178 | 0.07113601 | 0.39971663 |
| CCL4 | P13236 | -0.3907628 | -1.8153864 | -5.2977124 | 0.07709329 | 0.41350035 |
| SFRP1 | Q8N474 | 0.47787233 | 1.75327631 | -5.425684 | 0.08734458 | 0.43218854 |
| AB40 | P05067 | -0.2135349 | -1.7500739 | -5.4130267 | 0.08790275 | 0.43218854 |
| SAA1 | P0DJI8 | -0.7826509 | -1.7081421 | -5.5068087 | 0.0955022 | 0.44489002 |
| CXCL1 | P09341 | -0.8158079 | -1.6783764 | -5.53413 | 0.10121313 | 0.44489002 |
| VSNL1 | P62760 | 0.2017937 | 1.66764754 | -5.5548612 | 0.10332833 | 0.44489002 |
| IL18 | Q14116 | -0.8335711 | -1.6566034 | -5.5733952 | 0.10556712 | 0.44489002 |
| IL4 | P05112 | 0.4106815 | 1.6302057 | -5.5899864 | 0.11104025 | 0.45181894 |
| SFTPD | P35247 | -0.4708241 | -1.5932347 | -5.6798736 | 0.11911198 | 0.46850711 |
| CCL3 | P10147 | -0.2240979 | -1.557076 | -5.7147188 | 0.127462 | 0.48517794 |
| CD40LG | P29965 | -0.9439085 | -1.5386816 | -5.7516036 | 0.13189924 | 0.48637845 |
| KLK6 | Q92876 | -0.2698707 | -1.4571412 | -5.8718874 | 0.15301417 | 0.52724823 |
| SQSTM1 | Q13501 | -0.4533676 | -1.4321455 | -5.9269951 | 0.16000075 | 0.52724823 |
| NPTX1 | Q15818 | 0.25676751 | 1.42811868 | -5.9058745 | 0.16114929 | 0.52724823 |
| Oligo-SNCA | P37840 | -1.5345273 | -1.3964907 | -5.9733777 | 0.17040807 | 0.52724823 |
| SNCA | P37840 | -0.678714 | -1.3614023 | -6.0210992 | 0.18114484 | 0.52724823 |
| CXCL10 | P02778 | -0.3312861 | -1.3462096 | -6.0300861 | 0.18594152 | 0.52724823 |
| IGF1 | P05019 | -0.088904 | -1.34282 | -6.0106044 | 0.18702741 | 0.52724823 |
| TARDBP | Q13148 | -0.8103686 | -1.3326412 | -6.0469969 | 0.19032843 | 0.52724823 |
| IL16 | Q14005 | -0.2297843 | -1.3211567 | -6.0305779 | 0.19408257 | 0.52724823 |
| TIMP3 | P35625 | -0.8308845 | -1.3078341 | -6.0889362 | 0.19853186 | 0.52724823 |
| PSEN1 | P49768 | -0.6069135 | -1.3069576 | -6.0915412 | 0.19882654 | 0.52724823 |
| SOD1 | P00441 | -0.6800011 | -1.2812616 | -6.1269418 | 0.20761399 | 0.52724823 |
| REST | Q13127 | -0.280162 | -1.259675 | -6.1231066 | 0.21520909 | 0.52724823 |
| NRGN | Q92686 | -0.887243 | -1.2592835 | -6.1338483 | 0.21535951 | 0.52724823 |
| CCL11 | P51671 | -0.1382335 | -1.2497673 | -6.1349548 | 0.21876899 | 0.52724823 |
| FGF2 | P09038 | -0.7974367 | -1.2259427 | -6.1618929 | 0.22751911 | 0.52724823 |
| ANXA5 | P08758 | -1.7970464 | -1.2200398 | -6.2006368 | 0.2297239 | 0.52724823 |
| VCAM1 | P19320 | -0.1207762 | -1.2108478 | -6.1494776 | 0.23317831 | 0.52724823 |

**Table S5**- Comparison of plasma protein expression between LB+ and AD (first 50 results by *P* value).

| ID | UniProtID | logFC | *t* | *B* | *P* value | Adj. *P* value |
| --- | --- | --- | --- | --- | --- | --- |
| pTau217 | P10636 | -1.0047838 | -5.3469519 | 4.88240041 | 1.85E-06 | 0.00023 |
| ENO2 | P09104 | -2.0253943 | -3.7909443 | -0.1604672 | 0.00038025 | 0.02357531 |
| FLT1 | P17948 | 0.53761162 | 3.46830486 | -1.0716818 | 0.00103553 | 0.03627919 |
| AB42 | P05067 | 1.09261336 | 3.42818312 | -1.1956855 | 0.0011703 | 0.03627919 |
| ARSA | P15289 | -2.038832 | -2.8016165 | -2.8436017 | 0.00704447 | 0.16198751 |
| GDF15 | Q99988 | 0.66022631 | 2.76190445 | -2.9181001 | 0.00783811 | 0.16198751 |
| PRDX6 | P30041 | -0.9753834 | -2.5514974 | -3.422475 | 0.01359141 | 0.24076204 |
| pTau231 | P10636 | -0.4866509 | -2.4744796 | -3.5781179 | 0.01651641 | 0.25600435 |
| SNAP25 | P60880 | -0.2086949 | -2.3712431 | -3.7943353 | 0.02132086 | 0.27856811 |
| NEFL | P07196 | 0.35265903 | 2.34979164 | -3.8268429 | 0.02246517 | 0.27856811 |
| CRP | P02741 | 0.6537743 | 2.28922609 | -3.9849509 | 0.02600318 | 0.29312679 |
| IL18 | Q14116 | -0.5815769 | -2.1099585 | -4.3326573 | 0.03951239 | 0.40829467 |
| MAPT | P10636 | -0.2441972 | -2.0522002 | -4.4256978 | 0.04500542 | 0.41227096 |
| AB4240 | P123 | 0.08616613 | 2.01553027 | -4.5036612 | 0.04883152 | 0.41227096 |
| VCAM1 | P19320 | 0.21132311 | 2.00599295 | -4.5340051 | 0.04987149 | 0.41227096 |
| YWHAZ | P63104 | -1.2059419 | -1.959889 | -4.6416395 | 0.05517918 | 0.42763866 |
| pTau181 | P10636 | -0.2975587 | -1.7896198 | -4.9043227 | 0.07911666 | 0.57708626 |
| NGF | P01138 | 0.2251283 | 1.74388812 | -4.9978235 | 0.08686262 | 0.58217261 |
| BACE1 | P56817 | -0.179414 | -1.6800679 | -5.0885494 | 0.09871447 | 0.58217261 |
| SQSTM1 | Q13501 | 0.25623221 | 1.64891444 | -5.1286522 | 0.10496491 | 0.58217261 |
| SMOC1 | Q9H4F8 | 0.34704854 | 1.61786366 | -5.1791616 | 0.11152043 | 0.58217261 |
| GFAP | P14136 | -0.2371986 | -1.5629335 | -5.2414895 | 0.12390357 | 0.58217261 |
| CNTN2 | Q02246 | -0.6065268 | -1.5756608 | -5.2672476 | 0.12094639 | 0.58217261 |
| HBA1 | P69905 | -0.9833924 | -1.5310707 | -5.3106549 | 0.13159103 | 0.58217261 |
| PARK7 | Q99497 | -0.7625749 | -1.5358296 | -5.3279214 | 0.13042054 | 0.58217261 |
| VGF | O15240 | 0.43226121 | 1.46328724 | -5.3881867 | 0.1491869 | 0.58217261 |
| VEGFD | O43915 | -0.5093568 | -1.4863585 | -5.396016 | 0.14300127 | 0.58217261 |
| VEGFA | P15692 | 0.20600819 | 1.4777827 | -5.4005203 | 0.14526848 | 0.58217261 |
| SNCA | P37840 | -0.4116657 | -1.474916 | -5.4057004 | 0.14604335 | 0.58217261 |
| IL6 | P05231 | 0.31987947 | 1.46539557 | -5.4156621 | 0.14861306 | 0.58217261 |
| FABP3 | P05413 | 0.27040963 | 1.44332296 | -5.4507841 | 0.15470724 | 0.58217261 |
| TEK | Q02763 | -0.1919013 | -1.416749 | -5.4744597 | 0.1622932 | 0.58217261 |
| MME | P08473 | 0.64262 | 1.41740039 | -5.4757338 | 0.16211156 | 0.58217261 |
| CCL26 | Q9Y258 | -0.3571589 | -1.3920341 | -5.4989639 | 0.16962027 | 0.58217261 |
| IL33 | O95760 | 0.29206435 | 1.38665844 | -5.5371333 | 0.17124544 | 0.58217261 |
| ACHE | P22303 | -0.1645201 | -1.3339963 | -5.5373583 | 0.18779762 | 0.58217261 |
| OligoSNCA | P37840 | -1.0515005 | -1.3599916 | -5.560087 | 0.17948497 | 0.58217261 |
| MDH1 | P40925 | -0.4640556 | -1.3641328 | -5.5611348 | 0.17818591 | 0.58217261 |
| BASP1 | P80723 | -0.3815424 | -1.3438467 | -5.5626606 | 0.1846187 | 0.58217261 |
| VSNL1 | P62760 | -0.1542101 | -1.3375968 | -5.5760166 | 0.18662803 | 0.58217261 |
| AB40 | P05067 | 0.28378455 | 1.29690072 | -5.6362433 | 0.20018083 | 0.59727366 |
| PDLIM5 | Q96HC4 | -0.4878668 | -1.2856508 | -5.6596537 | 0.20405237 | 0.59727366 |
| PGK1 | P00558 | -0.6948746 | -1.2679032 | -5.6754541 | 0.21027347 | 0.59727366 |
| IL1B | P01584 | -0.5850009 | -1.2632272 | -5.6947103 | 0.21193581 | 0.59727366 |
| FCN2 | Q15485 | -0.1648655 | -1.1704628 | -5.7405484 | 0.24694852 | 0.63795034 |
| S100A12 | P80511 | -0.5804367 | -1.2146584 | -5.7452877 | 0.22978248 | 0.63317839 |
| KLK6 | Q92876 | 0.17853297 | 1.18727299 | -5.7509562 | 0.24031058 | 0.63795034 |
| CCL11 | P51671 | -0.1615475 | -1.1742101 | -5.7947862 | 0.24545749 | 0.63795034 |
| FGF2 | P09038 | 0.37052548 | 1.11718933 | -5.8336708 | 0.26886062 | 0.68038199 |
| CX3CL1 | P78423 | 0.13417834 | 1.08706529 | -5.8779875 | 0.28183094 | 0.69894074 |

**Table S6**- Comparison of CSF protein expression between *GRN*+ and *GRN*- (first 50 results by *P* value).

| ID | UniProtID | logFC | *t* | *B* | *P* value | Adj. *P* value |
| --- | --- | --- | --- | --- | --- | --- |
| NEFL | P07196 | 2.73999573 | 13.33713 | 26.9812752 | 2.71E-16 | 3.20E-14 |
| SQSTM1 | Q13501 | 0.99014751 | 6.35904918 | 6.83486723 | 1.49E-07 | 8.80E-06 |
| SNCA | P37840 | 1.4912041 | 5.87449166 | 5.30670677 | 7.15E-07 | 2.66E-05 |
| ENO2 | P09104 | 1.48817792 | 5.80322845 | 5.08370853 | 9.00E-07 | 2.66E-05 |
| PRDX6 | P30041 | 1.70946599 | 5.57849309 | 4.35816168 | 1.86E-06 | 4.39E-05 |
| VEGFA | P15692 | 0.71326848 | 5.15006491 | 2.96460501 | 7.35E-06 | 0.00014453 |
| NGF | P01138 | 0.85770198 | 4.93126155 | 2.26937617 | 1.48E-05 | 0.0002204 |
| TEK | Q02763 | 0.61394465 | 4.92737403 | 2.26687448 | 1.49E-05 | 0.0002204 |
| TARDBP | Q13148 | 1.34365493 | 4.89034535 | 2.16715099 | 1.69E-05 | 0.00022102 |
| MDH1 | P40925 | 1.60487394 | 4.70800008 | 1.60532541 | 3.00E-05 | 0.00035396 |
| SOD1 | P00441 | 1.46028807 | 4.63786288 | 1.37508823 | 3.74E-05 | 0.00040115 |
| Oligo-SNCA | P37840 | 2.44499383 | 4.50274101 | 0.95950581 | 5.71E-05 | 0.00051792 |
| MAPT | P10636 | 0.61624078 | 4.50409266 | 0.9595037 | 5.67E-05 | 0.00051792 |
| TNF | P01375 | 0.56781759 | 4.4758619 | 0.89624658 | 6.19E-05 | 0.00052169 |
| ICAM1 | P05362 | 0.56131256 | 4.42733463 | 0.7468318 | 7.20E-05 | 0.00056619 |
| IGF1 | P05019 | 0.34230636 | 4.22819863 | 0.15888177 | 0.00013294 | 0.00098047 |
| AGRN | O00468 | 0.513112 | 4.08047405 | -0.3123496 | 0.00020869 | 0.00144857 |
| NPTX2 | P47972 | 0.60528757 | 4.04636934 | -0.4040851 | 0.00023179 | 0.0015195 |
| NRGN | Q92686 | 2.54037953 | 3.92970647 | -0.7253821 | 0.00032919 | 0.00204444 |
| FGF2 | P09038 | 1.69408488 | 3.89065246 | -0.8444424 | 0.00036992 | 0.00218253 |
| POSTN | Q15063 | 0.82918783 | 3.84679494 | -0.9831263 | 0.00042149 | 0.00230036 |
| PSEN1 | P49768 | 0.83938926 | 3.82595449 | -1.0380619 | 0.00044838 | 0.00230036 |
| PGF | P49763 | 0.57767099 | 3.82661236 | -1.0384938 | 0.0004475 | 0.00230036 |
| CX3CL1 | P78423 | 0.52129281 | 3.78694778 | -1.1633699 | 0.00050266 | 0.00247143 |
| GOT1 | P17174 | 0.69474391 | 3.76565388 | -1.2241009 | 0.00053585 | 0.00252919 |
| IGFBP7 | Q16270 | 0.38361108 | 3.74449803 | -1.2781509 | 0.00056924 | 0.00258348 |
| CHI3L1 | P36222 | 1.02262047 | 3.67636805 | -1.4649181 | 0.00069629 | 0.00304306 |
| BASP1 | P80723 | 0.50186094 | 3.6276597 | -1.5770859 | 0.00080165 | 0.00319449 |
| ACHE | P22303 | 0.60647878 | 3.63627378 | -1.5873411 | 0.00078259 | 0.00319449 |
| PDGFRB | P09619 | 0.67876864 | 3.62350903 | -1.6021394 | 0.00081216 | 0.00319449 |
| BACE1 | P56817 | 0.32452041 | 3.55460704 | -1.7897848 | 0.00098975 | 0.00376745 |
| IL12p70 | P29459\|P29460 | 0.55844791 | 3.42835557 | -2.11444 | 0.00142243 | 0.00508625 |
| REST | Q13127 | 0.55894363 | 3.42907099 | -2.152675 | 0.00141954 | 0.00508625 |
| PGK 1.00 | P00558 | 1.91371995 | 3.41125983 | -2.1907724 | 0.00149308 | 0.00518186 |
| IL33 | O95760 | 0.38226355 | 3.383871 | -2.2509632 | 0.00161117 | 0.00543193 |
| TAFA5 | Q7Z5A7 | 0.81044807 | 3.32868128 | -2.3875114 | 0.00188424 | 0.00600918 |
| CST3 | P01034 | 0.28639869 | 3.34059337 | -2.387983 | 0.00181999 | 0.00596554 |
| FLT1 | P17948 | 0.60414088 | 3.21734853 | -2.6967181 | 0.00256822 | 0.007975 |
| NCAM1 | P13591 | 0.31458329 | 3.19869271 | -2.7651242 | 0.00270074 | 0.00817147 |
| CCL3 | P10147 | 0.546836 | 3.0969937 | -3.0138508 | 0.00356993 | 0.01036976 |
| SLIT2 | O94813 | 0.41105196 | 3.09330951 | -3.0181801 | 0.00360305 | 0.01036976 |
| CCL2 | P13500 | 0.66196008 | 3.07268321 | -3.0863569 | 0.00381276 | 0.01071203 |
| IL6 | P05231 | 0.62265564 | 2.83754319 | -3.6512411 | 0.00711139 | 0.01951497 |
| IL18 | Q14116 | 0.93413516 | 2.79055791 | -3.7698621 | 0.00803066 | 0.02153677 |
| CCL22 | O00626 | 0.5090017 | 2.7538799 | -3.8605024 | 0.00882374 | 0.0231378 |
| HBA1 | P69905 | 1.29706484 | 2.73826852 | -3.8670661 | 0.00918284 | 0.02355598 |
| IL10 | P22301 | 0.62938766 | 2.65986985 | -4.0859984 | 0.01119959 | 0.02811813 |
| CSF2 | P04141 | 0.42688577 | 2.57067587 | -4.262276 | 0.01398587 | 0.03438192 |
| IL1B | P01584 | 0.2324924 | 2.53880983 | -4.3223871 | 0.01511826 | 0.0349795 |
| KDR | P35968 | 0.49129996 | 2.5425078 | -4.3334952 | 0.0149898 | 0.0349795 |

**Table S7**- Correlation of the same protein targets in plasma and serum (ranked by correlation coefficient).

| Target | Cor. Coeff. | *P* value | Lower ci | Upper ci | Adj. *P* value |
| --- | --- | --- | --- | --- | --- |
| NEFH | 0.97144179 | 2.40E-25 | 0.94629328 | 0.98490568 | 2.72E-23 |
| MME | 0.96556016 | 8.02E-24 | 0.93540197 | 0.98177133 | 4.53E-22 |
| KDR | 0.9592461 | 1.86E-22 | 0.92377325 | 0.97839669 | 7.00E-21 |
| SAA1 | 0.95599473 | 7.77E-22 | 0.91781055 | 0.97665496 | 2.19E-20 |
| IL13 | 0.95024577 | 7.61E-21 | 0.9073095 | 0.97356864 | 1.72E-19 |
| IFNG | 0.94816492 | 1.63E-20 | 0.90352179 | 0.97244943 | 3.07E-19 |
| MSLN | 0.93620077 | 7.58E-19 | 0.88187824 | 0.96599259 | 1.22E-17 |
| CRP | 0.92889562 | 5.58E-18 | 0.8687746 | 0.96203177 | 7.88E-17 |
| IL6 | 0.92267275 | 2.60E-17 | 0.85767832 | 0.95864672 | 3.26E-16 |
| NEFL | 0.92071287 | 4.11E-17 | 0.85419606 | 0.95757849 | 4.64E-16 |
| IL17A | 0.91524077 | 1.39E-16 | 0.84450487 | 0.95459056 | 1.43E-15 |
| TREM2 | 0.90609853 | 8.98E-16 | 0.8284165 | 0.94958091 | 8.22E-15 |
| IL5 | 0.90582892 | 9.46E-16 | 0.82794399 | 0.94943284 | 8.22E-15 |
| CRH | 0.90183908 | 2.01E-15 | 0.8209643 | 0.94723926 | 1.62E-14 |
| GFAP | 0.88771691 | 2.27E-14 | 0.79645216 | 0.93944073 | 1.71E-13 |
| CHI3L1 | 0.88009901 | 7.39E-14 | 0.78335298 | 0.93521161 | 5.22E-13 |
| CSF2 | 0.87887537 | 8.86E-14 | 0.78125688 | 0.93453083 | 5.89E-13 |
| ACHE | 0.82787623 | 4.41E-11 | 0.6958102 | 0.90579026 | 2.77E-10 |
| IL12p70 | 0.82483342 | 5.98E-11 | 0.69082762 | 0.90405252 | 3.56E-10 |
| VSNL1 | 0.81902663 | 1.05E-10 | 0.68135422 | 0.90072898 | 5.95E-10 |
| IL2 | 0.809582 | 2.53E-10 | 0.66604381 | 0.89530282 | 1.36E-09 |
| PDGFRB | 0.79996525 | 5.91E-10 | 0.65057768 | 0.8897515 | 3.03E-09 |
| TREM1 | 0.79227276 | 1.12E-09 | 0.63829481 | 0.88529177 | 5.53E-09 |
| FABP3 | 0.78351238 | 2.27E-09 | 0.62440163 | 0.88019201 | 1.07E-08 |
| PTN | 0.78195794 | 2.56E-09 | 0.6219469 | 0.87928477 | 1.16E-08 |
| CHIT1 | 0.78015689 | 7.72E-09 | 0.61347653 | 0.8802925 | 3.12E-08 |
| NGF | 0.77924952 | 3.16E-09 | 0.61767733 | 0.87770232 | 1.37E-08 |
| FOLR1 | 0.77339362 | 4.91E-09 | 0.60847851 | 0.87427356 | 2.06E-08 |
| CXCL10 | 0.75355909 | 2.00E-08 | 0.577647 | 0.8625847 | 7.80E-08 |
| GDNF | 0.75185307 | 2.24E-08 | 0.57501834 | 0.86157384 | 8.45E-08 |
| BACE1 | 0.74680821 | 3.13E-08 | 0.56726644 | 0.85857955 | 1.14E-07 |
| CCL4 | 0.73992512 | 4.88E-08 | 0.55674101 | 0.85448187 | 1.72E-07 |
| CCL22 | 0.7240932 | 1.29E-07 | 0.53275245 | 0.8450023 | 4.40E-07 |
| PDLIM5 | 0.71019799 | 2.85E-07 | 0.51194876 | 0.83661928 | 9.49E-07 |
| CCL3 | 0.70123551 | 4.66E-07 | 0.49865253 | 0.83118059 | 1.50E-06 |
| IL10 | 0.69972653 | 5.05E-07 | 0.49642323 | 0.83026245 | 1.59E-06 |
| CALB2 | 0.67150919 | 2.10E-06 | 0.4552243 | 0.81296202 | 6.42E-06 |
| IL33 | 0.66392696 | 3.01E-06 | 0.44430944 | 0.80827029 | 8.94E-06 |
| pTau217 | 0.66281686 | 3.16E-06 | 0.44271688 | 0.80758184 | 9.17E-06 |
| IL9 | 0.65910939 | 3.75E-06 | 0.43740813 | 0.80527972 | 1.06E-05 |
| FCN2 | 0.6503401 | 5.56E-06 | 0.42491251 | 0.79981692 | 1.53E-05 |
| AGRN | 0.63865018 | 9.23E-06 | 0.40838773 | 0.79249601 | 2.48E-05 |
| FGF2 | 0.62903039 | 1.38E-05 | 0.39490154 | 0.7864381 | 3.56E-05 |
| SFTPD | 0.62889711 | 1.39E-05 | 0.3947154 | 0.78635395 | 3.56E-05 |
| IL4 | 0.62551362 | 1.59E-05 | 0.38999636 | 0.78421588 | 3.99E-05 |
| CCL2 | 0.61275833 | 2.64E-05 | 0.37231679 | 0.77612162 | 6.44E-05 |
| ICAM1 | 0.61234827 | 2.68E-05 | 0.37175131 | 0.77586051 | 6.44E-05 |
| CD63 | 0.61079345 | 2.84E-05 | 0.36960878 | 0.77486995 | 6.56E-05 |
| PGF | 0.61076466 | 2.85E-05 | 0.36956913 | 0.7748516 | 6.56E-05 |
| NPTX2 | 0.60419853 | 3.65E-05 | 0.36054945 | 0.77065939 | 8.26E-05 |
| IL15 | 0.59881642 | 4.47E-05 | 0.35319006 | 0.76721235 | 9.89E-05 |
| CCL17 | 0.59775868 | 4.64E-05 | 0.35174729 | 0.76653376 | 0.00010091 |
| VCAM1 | 0.58901312 | 6.38E-05 | 0.33986285 | 0.7609086 | 0.00013598 |
| TIMP3 | 0.58429396 | 7.54E-05 | 0.33348284 | 0.75786247 | 0.0001578 |
| SLIT2 | 0.57770305 | 9.49E-05 | 0.3246106 | 0.75359544 | 0.00019492 |
| CXCL8 | 0.5541335 | 0.00020744 | 0.29324312 | 0.738214 | 0.00041858 |
| CCL26 | 0.52818164 | 0.00046002 | 0.25934288 | 0.72105351 | 0.00091196 |
| TNF | 0.51981434 | 0.00058678 | 0.24855233 | 0.71546979 | 0.00114321 |
| IGFBP7 | 0.51471128 | 0.0006786 | 0.24200425 | 0.71205207 | 0.0012997 |
| VEGFA | 0.50694645 | 0.00084299 | 0.23208812 | 0.70683365 | 0.00158763 |
| HBA1 | 0.49716858 | 0.00174533 | 0.2063988 | 0.70725449 | 0.00308159 |
| NPTX1 | 0.49463356 | 0.00117684 | 0.21648011 | 0.69851382 | 0.00218005 |
| S100B | 0.48740347 | 0.00142338 | 0.20738092 | 0.69360262 | 0.00259423 |
| CX3CL1 | 0.48469168 | 0.00152702 | 0.20398053 | 0.69175562 | 0.00273893 |
| IGF1 | 0.45916313 | 0.0028797 | 0.17229821 | 0.67423431 | 0.00500624 |
| SNAP25 | 0.45406177 | 0.00325038 | 0.16603752 | 0.67070377 | 0.00556504 |
| IL16 | 0.43993198 | 0.00450305 | 0.14881694 | 0.66087331 | 0.0075947 |
| CCL11 | 0.43659183 | 0.00485421 | 0.1447718 | 0.65853836 | 0.00806655 |
| SFRP1 | 0.4357697 | 0.0049442 | 0.14377764 | 0.65796299 | 0.00809702 |
| S100A12 | 0.42268503 | 0.00658407 | 0.12803395 | 0.64877054 | 0.01062858 |
| IL6R | 0.41126183 | 0.00838003 | 0.11440973 | 0.64069103 | 0.01333724 |
| PGK1 | 0.37350876 | 0.01759645 | 0.07016297 | 0.61362226 | 0.02761665 |
| AB40 | 0.37200266 | 0.01809462 | 0.06842223 | 0.61253055 | 0.02800948 |
| TEK | 0.36976904 | 0.01885521 | 0.06584404 | 0.61090982 | 0.02879242 |
| CNTN2 | 0.36777265 | 0.01955756 | 0.06354309 | 0.6094595 | 0.02946673 |
| CCL13 | 0.3635204 | 0.02112686 | 0.05865291 | 0.60636498 | 0.0314123 |
| GOT1 | 0.33565882 | 0.03422263 | 0.02696941 | 0.58590539 | 0.05022281 |
| NPY | 0.32696628 | 0.03946292 | 0.0172098 | 0.57945624 | 0.05717064 |
| NPTXR | 0.30425616 | 0.05628854 | -0.0080126 | 0.56245663 | 0.08051399 |
| CXCL1 | 0.28717095 | 0.07238445 | -0.0267295 | 0.54952202 | 0.10224303 |
| VEGFD | 0.28063726 | 0.0794194 | -0.0338295 | 0.54454212 | 0.11079497 |
| KLK6 | 0.27698242 | 0.08358104 | -0.0377873 | 0.54174831 | 0.11517875 |
| TAFA5 | 0.26712102 | 0.09565714 | -0.0484172 | 0.53418081 | 0.130232 |
| IL18 | 0.26220657 | 0.10215506 | -0.053688 | 0.53039348 | 0.13742288 |
| ENO2 | 0.25202003 | 0.11669121 | -0.0645575 | 0.52250898 | 0.15495072 |
| RUVBL2 | 0.2511992 | 0.1179271 | -0.0654301 | 0.52187162 | 0.15495072 |
| FLT1 | 0.24569893 | 0.12646422 | -0.0712649 | 0.517593 | 0.16425812 |
| PARK7 | 0.23858344 | 0.13818309 | -0.078781 | 0.51203765 | 0.17743965 |
| CST3 | 0.19082174 | 0.23821146 | -0.128315 | 0.47414652 | 0.29908772 |
| pTau231 | 0.1872458 | 0.24728784 | -0.1319607 | 0.47126672 | 0.30707171 |
| POSTN | 0.17635999 | 0.27633493 | -0.1430058 | 0.46246267 | 0.33412186 |
| AB38 | 0.17213395 | 0.28818942 | -0.1472723 | 0.45902952 | 0.34279374 |
| MAPT | 0.16136772 | 0.31985523 | -0.1580883 | 0.45024438 | 0.37649626 |
| ANXA5 | 0.15460985 | 0.34080506 | -0.1648385 | 0.44470131 | 0.39702033 |
| MDH1 | 0.14135384 | 0.38428306 | -0.1779931 | 0.43376327 | 0.4431019 |
| IL1B | 0.12170762 | 0.45438432 | -0.1972813 | 0.41739235 | 0.51864069 |
| AB42 | 0.1007191 | 0.53632501 | -0.217618 | 0.39968825 | 0.60604726 |
| SQSTM1 | 0.06304686 | 0.69913819 | -0.25344 | 0.36734173 | 0.75964053 |
| SOD1 | 0.05546934 | 0.73388939 | -0.260542 | 0.36074529 | 0.78235378 |
| SNCA | 0.02428046 | 0.8817782 | -0.2894179 | 0.3332691 | 0.9312237 |
| pTau181 | 0.01962624 | 0.90432262 | -0.2936786 | 0.32912341 | 0.94374293 |
| UCHL1 | 0.01838751 | 0.9103361 | -0.2948105 | 0.32801801 | 0.94374293 |
| pTDP43 | 0.00504688 | 0.9753435 | -0.306945 | 0.31605933 | 0.98648385 |
| OligoSNCA | 0.0042233 | 0.97936615 | -0.3076908 | 0.31531782 | 0.98648385 |
| REST | -0.0027663 | 0.98648385 | -0.3140051 | 0.30900934 | 0.98648385 |
| BASP1 | -0.0107232 | 0.94763982 | -0.3211598 | 0.30179423 | 0.97348455 |
| TARDBP | -0.0583578 | 0.7205766 | -0.3632634 | 0.25783883 | 0.77547768 |
| IL7 | -0.0694144 | 0.67039354 | -0.3728613 | 0.24744554 | 0.73548029 |
| PRDX6 | -0.0755482 | 0.64313459 | -0.378158 | 0.24164809 | 0.71249224 |
| CD40LG | -0.079988 | 0.62368523 | -0.3819796 | 0.23743759 | 0.69778644 |
| YWHAZ | -0.1757802 | 0.27794208 | -0.4619922 | 0.14359182 | 0.33412186 |
| PSEN1 | -0.1764452 | 0.2760993 | -0.4625318 | 0.14291965 | 0.33412186 |
| NRGN | -0.2273679 | 0.15825663 | -0.5032345 | 0.09055524 | 0.20093257 |

**Table S8**- Correlation of the same protein targets in CSF and plasma (ranked by correlation coefficient).

| Target | Cor. Coeff. | *P* value | Lower CI | Upper CI | Adj. *P* value |
| --- | --- | --- | --- | --- | --- |
| PDGFRB | 0.72839218 | 9.95E-08 | 0.539236 | 0.84758391 | 5.77E-06 |
| pTau217 | 0.72134391 | 1.51E-07 | 0.52861782 | 0.84334835 | 5.84E-06 |
| NEFL | 0.70037798 | 4.88E-07 | 0.49738531 | 0.83065891 | 1.42E-05 |
| IL13 | 0.67194423 | 2.06E-06 | 0.45585252 | 0.81323065 | 4.63E-05 |
| CCL4 | 0.66879124 | 2.39E-06 | 0.45130423 | 0.81128231 | 4.63E-05 |
| ACHE | 0.64338965 | 7.54E-06 | 0.41506927 | 0.7954695 | 0.0001249 |
| CCL17 | 0.61175965 | 2.74E-05 | 0.37093989 | 0.7754856 | 0.00039716 |
| KDR | 0.59565952 | 7.97E-05 | 0.34088909 | 0.76892273 | 0.00102691 |
| CHIT1 | 0.58616974 | 0.00011019 | 0.32793833 | 0.76289516 | 0.00116202 |
| TREM2 | 0.57899511 | 9.07E-05 | 0.32634638 | 0.7544331 | 0.0010525 |
| CCL13 | 0.5450697 | 0.00027599 | 0.2813283 | 0.73224758 | 0.00266794 |
| CSF2 | 0.53998036 | 0.00032285 | 0.27467356 | 0.72888478 | 0.00288079 |
| SAA1 | 0.50627298 | 0.00138824 | 0.21801813 | 0.71328348 | 0.01150259 |
| MSLN | 0.45132977 | 0.00346554 | 0.16269415 | 0.66880896 | 0.02680016 |
| pTau231 | 0.44030954 | 0.00446479 | 0.14927481 | 0.66113699 | 0.0323697 |
| SFTPD | 0.42655305 | 0.00605646 | 0.13267261 | 0.65149485 | 0.04132643 |
| ICAM1 | 0.41964116 | 0.00702671 | 0.12439269 | 0.64662261 | 0.04528327 |
| IL1B | 0.39822346 | 0.01092907 | 0.0989944 | 0.63140661 | 0.06338858 |
| VSNL1 | 0.39244988 | 0.01225348 | 0.09221384 | 0.62727386 | 0.06768589 |
| CCL22 | 0.35918836 | 0.02283249 | 0.05368596 | 0.6032048 | 0.1203895 |
| CCL3 | 0.34997819 | 0.02684222 | 0.04317595 | 0.59646053 | 0.13537815 |
| CXCL8 | 0.34616704 | 0.028664 | 0.0388467 | 0.59365955 | 0.13854267 |
| IL18 | 0.34265267 | 0.03043333 | 0.03486477 | 0.59107136 | 0.1404541 |
| CCL11 | 0.34065025 | 0.03148109 | 0.03260031 | 0.58959436 | 0.1404541 |
| SFRP1 | 0.32153308 | 0.04305798 | 0.01113946 | 0.57540913 | 0.17838304 |
| IL6R | 0.31469804 | 0.04795315 | 0.00353526 | 0.57030009 | 0.18802138 |
| KLK6 | 0.31380289 | 0.04862622 | 0.00254203 | 0.56962951 | 0.18802138 |
| GDNF | 0.28337528 | 0.07640905 | -0.030858 | 0.54663128 | 0.28591774 |
| IL33 | 0.27819558 | 0.08218128 | -0.0364747 | 0.54267631 | 0.29790715 |
| IGFBP7 | 0.26660926 | 0.09631859 | -0.0489669 | 0.53378692 | 0.33857444 |
| IL12p70 | 0.26202111 | 0.10240671 | -0.0538866 | 0.53025035 | 0.34938761 |
| IL9 | 0.25712046 | 0.10922953 | -0.0591245 | 0.52646256 | 0.35977545 |
| CCL26 | 0.25494988 | 0.11235942 | -0.0614389 | 0.52478147 | 0.35977545 |
| CST3 | 0.24596487 | 0.12604111 | -0.0709832 | 0.51780018 | 0.38475706 |
| BDNF | 0.22986365 | 0.3747926 | -0.2819295 | 0.6398194 | 0.65872638 |
| SNCA | 0.22582065 | 0.16118401 | -0.0921726 | 0.50201559 | 0.47722145 |
| IL10 | 0.22276175 | 0.16708668 | -0.0953652 | 0.4996025 | 0.47722145 |
| ANXA5 | 0.21727081 | 0.17807062 | -0.1010798 | 0.49526002 | 0.48037655 |
| NGF | 0.20990132 | 0.19360978 | -0.1087166 | 0.48940999 | 0.51042578 |
| VEGFD | 0.20436873 | 0.20588716 | -0.1144252 | 0.48500154 | 0.52687424 |
| SOD1 | 0.20303211 | 0.20893289 | -0.1158012 | 0.48393435 | 0.52687424 |
| GOT1 | 0.19644955 | 0.22438928 | -0.12256 | 0.47866648 | 0.55381183 |
| SQSTM1 | 0.18999842 | 0.24028092 | -0.1291551 | 0.47348401 | 0.58067888 |
| IL6 | 0.18659595 | 0.2489619 | -0.1326222 | 0.47074273 | 0.5893792 |
| IL7 | 0.17741645 | 0.27342205 | -0.1419373 | 0.46331957 | 0.61226921 |
| NEFH | 0.17674719 | 0.275265 | -0.1426143 | 0.46277679 | 0.61226921 |
| PRDX6 | 0.17117739 | 0.29091765 | -0.1482364 | 0.45825125 | 0.61226921 |
| CXCL10 | 0.16955663 | 0.29557824 | -0.1498686 | 0.45693155 | 0.61226921 |
| FCN2 | 0.16542297 | 0.39112691 | -0.2140609 | 0.50152213 | 0.66059453 |
| PSEN1 | 0.16436428 | 0.31083036 | -0.1550856 | 0.45269517 | 0.62848144 |
| SLIT2 | 0.16322555 | 0.31424072 | -0.1562274 | 0.45176436 | 0.62848144 |
| IFNG | 0.1572036 | 0.33266665 | -0.1622513 | 0.44683144 | 0.65017623 |
| NRGN | 0.15299089 | 0.3459464 | -0.1664512 | 0.44337006 | 0.65017623 |
| NPTX2 | 0.14972724 | 0.35645397 | -0.169697 | 0.44068252 | 0.65017623 |
| TREM1 | 0.14814639 | 0.36161229 | -0.1712667 | 0.43937885 | 0.65017623 |
| FLT1 | 0.14732112 | 0.36432289 | -0.1720856 | 0.43869779 | 0.65017623 |
| RUVBL2 | 0.14100831 | 0.38545815 | -0.1783344 | 0.43347701 | 0.66059453 |
| VEGFA | 0.13172829 | 0.41779978 | -0.187474 | 0.42576649 | 0.69235392 |
| IL16 | 0.12823032 | 0.43037715 | -0.1909047 | 0.42284899 | 0.69338541 |
| PDLIM5 | 0.12310389 | 0.44918523 | -0.1959185 | 0.41856221 | 0.71377379 |
| GFAP | 0.11591049 | 0.47631462 | -0.2029259 | 0.41252473 | 0.72700652 |
| S100A12 | 0.11315023 | 0.48694876 | -0.2056062 | 0.41020108 | 0.73358515 |
| ENO2 | 0.1058797 | 0.51553764 | -0.2126432 | 0.40406206 | 0.74761097 |
| NCAM1 | 0.10315529 | 0.52646158 | -0.2152716 | 0.40175472 | 0.74761097 |
| BACE1 | 0.09917464 | 0.54262392 | -0.2191037 | 0.39837661 | 0.74761097 |
| TNF | 0.09819959 | 0.54661875 | -0.2200408 | 0.39754791 | 0.74761097 |
| CXCL1 | 0.09634498 | 0.55425547 | -0.2218218 | 0.39597032 | 0.74761097 |
| pTau181 | 0.09319583 | 0.56733631 | -0.224841 | 0.39328751 | 0.75644841 |
| AB42 | 0.0904381 | 0.57890651 | -0.2274799 | 0.39093394 | 0.76310403 |
| FOLR1 | 0.08263489 | 0.6122076 | -0.2349217 | 0.38425297 | 0.79793349 |
| NPY | 0.07762713 | 0.63399719 | -0.239678 | 0.37994872 | 0.79938776 |
| CRH | 0.06880548 | 0.67312312 | -0.2480198 | 0.37233438 | 0.80722862 |
| MDH1 | 0.06686372 | 0.6818551 | -0.2498497 | 0.37065286 | 0.80722862 |
| CX3CL1 | 0.05701381 | 0.72676106 | -0.2590972 | 0.36209228 | 0.85155842 |
| CD63 | 0.05210063 | 0.74951312 | -0.2636883 | 0.3578029 | 0.85159758 |
| TEK | 0.05067401 | 0.75615992 | -0.2650188 | 0.35655499 | 0.85159758 |
| POSTN | 0.04347387 | 0.78996196 | -0.2717152 | 0.35024008 | 0.87271988 |
| CCL2 | 0.03359538 | 0.83695011 | -0.2808532 | 0.34153052 | 0.90533839 |
| OligoSNCA | 0.03235335 | 0.84290126 | -0.2819981 | 0.3404317 | 0.90533839 |
| IL2 | 0.01868695 | 0.908882 | -0.2945369 | 0.3282853 | 0.9248273 |
| IL5 | 0.00613323 | 0.97003831 | -0.3059607 | 0.31703686 | 0.97847343 |
| CHI3L1 | 0.0011248 | 0.99450402 | -0.3104933 | 0.31252464 | 0.99450402 |
| IL17A | -0.0226057 | 0.88988106 | -0.3317787 | 0.29095254 | 0.91350622 |
| CALB2 | -0.0243567 | 0.88140969 | -0.3333369 | 0.28934805 | 0.9128886 |
| IGF1 | -0.0263092 | 0.8719791 | -0.3350724 | 0.28755688 | 0.91125744 |
| PGF | -0.0267973 | 0.86962409 | -0.335506 | 0.28710874 | 0.91125744 |
| SNAP25 | -0.0287379 | 0.86027246 | -0.3372284 | 0.2853257 | 0.91125744 |
| YWHAZ | -0.0329417 | 0.84008118 | -0.3409523 | 0.28145586 | 0.90533839 |
| MAPT | -0.0474761 | 0.77112196 | -0.3537537 | 0.26799675 | 0.86009757 |
| PGK1 | -0.0507806 | 0.75566254 | -0.3566483 | 0.26491938 | 0.85159758 |
| PARK7 | -0.0528689 | 0.74594085 | -0.3584745 | 0.26297133 | 0.85159758 |
| S100B | -0.0668385 | 0.681969 | -0.370631 | 0.24987346 | 0.80722862 |
| IL15 | -0.0702249 | 0.66676669 | -0.3735623 | 0.24668078 | 0.80722862 |
| CNTN2 | -0.0712162 | 0.66234091 | -0.3744192 | 0.24574488 | 0.80722862 |
| REST | -0.0760107 | 0.64109718 | -0.3785566 | 0.24121002 | 0.7996481 |
| AGRN | -0.0777334 | 0.63353149 | -0.3800402 | 0.23957723 | 0.79938776 |
| YWHAG | -0.0797638 | 0.62466141 | -0.3817868 | 0.23765048 | 0.79938776 |
| TIMP3 | -0.1004535 | 0.53740568 | -0.3994628 | 0.21787359 | 0.74761097 |
| PTN | -0.1016912 | 0.53237857 | -0.4005132 | 0.21668215 | 0.74761097 |
| UCHL1 | -0.1054309 | 0.51732943 | -0.4036822 | 0.21307647 | 0.74761097 |
| HBA1 | -0.108552 | 0.55426331 | -0.4405698 | 0.24958927 | 0.74761097 |
| NPTXR | -0.1172574 | 0.47117024 | -0.4136572 | 0.20161634 | 0.72700652 |
| MME | -0.1200137 | 0.46073539 | -0.4159717 | 0.19893287 | 0.72223385 |
| FGF2 | -0.1294474 | 0.42597737 | -0.4238648 | 0.18971194 | 0.69338541 |
| CD40LG | -0.1446702 | 0.39293985 | -0.4477027 | 0.1881694 | 0.66059453 |
| VCAM1 | -0.1480879 | 0.3618039 | -0.4393306 | 0.17132476 | 0.65017623 |
| AB40 | -0.1497321 | 0.35643805 | -0.4406866 | 0.16969215 | 0.65017623 |
| IL4 | -0.1710268 | 0.29134857 | -0.4581287 | 0.14838811 | 0.61226921 |
| NPTX1 | -0.1744857 | 0.28155249 | -0.4609411 | 0.1448995 | 0.61226921 |
| pTDP43 | -0.1817864 | 0.26158793 | -0.4668584 | 0.13750988 | 0.60688399 |
| AB38 | -0.2198808 | 0.17278708 | -0.4973258 | 0.09836614 | 0.47722145 |
| BASP1 | -0.2200334 | 0.1724817 | -0.4974465 | 0.09820734 | 0.47722145 |
| FABP3 | -0.2533186 | 0.11475596 | -0.5235167 | 0.06317601 | 0.35977545 |
| TAFA5 | -0.3256021 | 0.04034174 | -0.5784412 | -0.0156835 | 0.17332006 |
| TARDBP | -0.3986953 | 0.01082645 | -0.6317438 | -0.0995497 | 0.06338858 |
| CRP | -0.8661632 | 5.26E-13 | -0.9274342 | -0.7596104 | 6.10E-11 |

**Table S9**- Correlation of the same protein targets in CSF and serum (ranked by correlation coefficient).

| Target | Cor. Coeff. | *P* value | Lower CI | Upper CI | Adj. *P* value |
| --- | --- | --- | --- | --- | --- |
| NEFL | 0.68192383 | 1.27E-06 | 0.47032319 | 0.81937658 | 5.64E-05 |
| ACHE | 0.67850484 | 1.50E-06 | 0.46535272 | 0.81727455 | 5.64E-05 |
| IL13 | 0.66563546 | 2.78E-06 | 0.44676321 | 0.80932908 | 7.84E-05 |
| PDGFRB | 0.63481362 | 1.08E-05 | 0.4029971 | 0.79008363 | 0.00024516 |
| KDR | 0.6216126 | 3.11E-05 | 0.37680829 | 0.78525142 | 0.00058541 |
| CCL4 | 0.59384732 | 5.36E-05 | 0.34642228 | 0.76402117 | 0.00086489 |
| CCL17 | 0.55233458 | 0.00021968 | 0.29087188 | 0.73703211 | 0.00310294 |
| pTau217 | 0.53994779 | 0.00032317 | 0.27463105 | 0.72886323 | 0.00405757 |
| SAA1 | 0.52331151 | 0.00088898 | 0.23998508 | 0.72448479 | 0.01004543 |
| CSF2 | 0.49123085 | 0.00128771 | 0.21219171 | 0.69620484 | 0.01322831 |
| TREM2 | 0.47249991 | 0.00207988 | 0.18877617 | 0.6834182 | 0.0195855 |
| CCL22 | 0.45408809 | 0.00324836 | 0.16606976 | 0.67072201 | 0.02823575 |
| CCL13 | 0.39135413 | 0.01251966 | 0.09093011 | 0.62648803 | 0.10105153 |
| MSLN | 0.37873346 | 0.01595678 | 0.076216 | 0.61740229 | 0.12020772 |
| CHIT1 | 0.37640732 | 0.0198477 | 0.06448332 | 0.62132582 | 0.14017435 |
| ICAM1 | 0.32442981 | 0.04110957 | 0.01437304 | 0.57756841 | 0.25807674 |
| IL12p70 | 0.31117868 | 0.05064334 | -0.0003662 | 0.56766171 | 0.28613488 |
| SFRP1 | 0.30368316 | 0.05677748 | -0.0086439 | 0.56202487 | 0.30551692 |
| REST | 0.29223871 | 0.06727287 | -0.0212006 | 0.55337181 | 0.33051453 |
| pTau231 | 0.25437922 | 0.11319346 | -0.0620468 | 0.52433915 | 0.45723328 |
| IL33 | 0.25240574 | 0.11611386 | -0.0641473 | 0.52280837 | 0.45723328 |
| S100B | 0.23957603 | 0.13650183 | -0.0777347 | 0.51281398 | 0.51415688 |
| NGF | 0.23642786 | 0.14188692 | -0.0810508 | 0.51035018 | 0.51720069 |
| NEFH | 0.22111203 | 0.17033405 | -0.0970843 | 0.49829929 | 0.58445927 |
| CCL3 | 0.21835844 | 0.175855 | -0.0999495 | 0.49612127 | 0.58445927 |
| GFAP | 0.20293964 | 0.20914474 | -0.1158964 | 0.4838605 | 0.62524912 |
| FOLR1 | 0.20245361 | 0.21026077 | -0.1163964 | 0.48347221 | 0.62524912 |
| IGFBP7 | 0.19871957 | 0.21897295 | -0.1202325 | 0.48048543 | 0.63446009 |
| IL10 | 0.19502446 | 0.22783615 | -0.1240193 | 0.47752334 | 0.64363712 |
| GDNF | 0.18837789 | 0.2443896 | -0.1308074 | 0.47217908 | 0.6648818 |
| POSTN | 0.18153362 | 0.26226307 | -0.1377663 | 0.46665396 | 0.6648818 |
| IL18 | 0.17865017 | 0.27004605 | -0.1406887 | 0.46431958 | 0.6648818 |
| CCL2 | 0.17666191 | 0.27550041 | -0.1427005 | 0.46270761 | 0.6648818 |
| CNTN2 | 0.17410002 | 0.28263406 | -0.1452889 | 0.46062778 | 0.6648818 |
| SFTPD | 0.16971396 | 0.29512373 | -0.1497102 | 0.45705971 | 0.6648818 |
| AB38 | 0.16800682 | 0.3000794 | -0.1514276 | 0.45566845 | 0.6648818 |
| VSNL1 | 0.1634048 | 0.31370232 | -0.1560477 | 0.45191093 | 0.66639117 |
| IFNG | 0.16182997 | 0.31845242 | -0.1576255 | 0.45062273 | 0.66639117 |
| IL6 | 0.15701231 | 0.33326272 | -0.1624422 | 0.44667445 | 0.67406561 |
| CALB2 | 0.15307312 | 0.34568413 | -0.1663693 | 0.44343771 | 0.67406561 |
| OligoSNCA | 0.14946291 | 0.35731336 | -0.1699596 | 0.44046463 | 0.67406561 |
| IL6R | 0.14936496 | 0.35763214 | -0.1700569 | 0.44038387 | 0.67406561 |
| IL17A | 0.14927934 | 0.35791094 | -0.1701419 | 0.44031328 | 0.67406561 |
| BACE1 | 0.14699985 | 0.3653814 | -0.1724042 | 0.43843257 | 0.67685406 |
| CCL11 | 0.12666065 | 0.43608887 | -0.1924417 | 0.42153782 | 0.74706096 |
| TNF | 0.12565526 | 0.43976922 | -0.1934253 | 0.42069735 | 0.74706096 |
| pTau181 | 0.12517873 | 0.44151961 | -0.1938913 | 0.4202988 | 0.74706096 |
| TARDBP | 0.12333611 | 0.44832368 | -0.1956918 | 0.41875668 | 0.74706096 |
| CRH | 0.12300327 | 0.44955881 | -0.1960168 | 0.41847793 | 0.74706096 |
| NPTX1 | 0.11606813 | 0.47571101 | -0.2027727 | 0.41265732 | 0.75711753 |
| IL1B | 0.10223663 | 0.53017048 | -0.2161568 | 0.40097583 | 0.7987902 |
| AB40 | 0.09668844 | 0.55283743 | -0.2214921 | 0.39626261 | 0.80090551 |
| TEK | 0.0912221 | 0.57560639 | -0.2267301 | 0.39160344 | 0.82333572 |
| IL15 | 0.07957008 | 0.62550555 | -0.2378344 | 0.38162027 | 0.86197715 |
| IL16 | 0.07678925 | 0.63767351 | -0.2404723 | 0.37922727 | 0.86815791 |
| CST3 | 0.06193518 | 0.70420071 | -0.254484 | 0.36637589 | 0.90538699 |
| CCL26 | 0.05559955 | 0.73328756 | -0.2604202 | 0.3608589 | 0.91923667 |
| MAPT | 0.05194379 | 0.75024304 | -0.2638347 | 0.35766575 | 0.91923667 |
| PDLIM5 | 0.05075696 | 0.75577296 | -0.2649414 | 0.35662758 | 0.91923667 |
| CX3CL1 | 0.04993238 | 0.75962216 | -0.2657099 | 0.35590583 | 0.91923667 |
| TREM1 | 0.04991359 | 0.75970994 | -0.2657274 | 0.35588938 | 0.91923667 |
| NPTX2 | 0.0463541 | 0.77639097 | -0.2690401 | 0.35276955 | 0.91923667 |
| KLK6 | 0.04628845 | 0.77669955 | -0.2691011 | 0.35271195 | 0.91923667 |
| IL9 | 0.04281944 | 0.7930542 | -0.2723223 | 0.34966473 | 0.91923667 |
| CXCL10 | 0.03666505 | 0.82228086 | -0.2780197 | 0.34424263 | 0.92228022 |
| SQSTM1 | 0.02894455 | 0.85927783 | -0.2851357 | 0.33741163 | 0.94270287 |
| VEGFD | 0.01954631 | 0.90471052 | -0.2937517 | 0.32905211 | 0.96006429 |
| PTN | 0.01305241 | 0.93628791 | -0.2996754 | 0.32324745 | 0.97475237 |
| PGF | 0.01156865 | 0.94351813 | -0.3010255 | 0.32191789 | 0.97475237 |
| NPTXR | 0.00793134 | 0.96126018 | -0.3043299 | 0.3186534 | 0.97475237 |
| CHI3L1 | 0.00693448 | 0.96612624 | -0.3052342 | 0.31775742 | 0.97475237 |
| NPY | -0.002645 | 0.98707634 | -0.3138957 | 0.30911903 | 0.98707634 |
| AB42 | -0.009068 | 0.9557133 | -0.3196744 | 0.30329807 | 0.97475237 |
| IL7 | -0.0186446 | 0.90908742 | -0.3282475 | 0.29457559 | 0.96006429 |
| pTDP43 | -0.023328 | 0.8863849 | -0.3324217 | 0.29029086 | 0.95391898 |
| VEGFA | -0.0254674 | 0.87604256 | -0.3343245 | 0.28832932 | 0.95185394 |
| AGRN | -0.0289643 | 0.85918268 | -0.3374292 | 0.28511755 | 0.94270287 |
| NRGN | -0.0362337 | 0.82433896 | -0.3438618 | 0.27841824 | 0.92228022 |
| IL2 | -0.0404873 | 0.80409898 | -0.3476125 | 0.27448392 | 0.91923667 |
| CXCL8 | -0.0415015 | 0.7992911 | -0.3485053 | 0.27354428 | 0.91923667 |
| FCN2 | -0.0478392 | 0.80534894 | -0.4072052 | 0.32435351 | 0.91923667 |
| ENO2 | -0.0617423 | 0.70508013 | -0.3662083 | 0.25466506 | 0.90538699 |
| IL5 | -0.0682256 | 0.67572644 | -0.3718324 | 0.24856651 | 0.88787311 |
| S100A12 | -0.0704741 | 0.66565279 | -0.3737778 | 0.24644551 | 0.88492666 |
| MME | -0.0739141 | 0.65035357 | -0.3767488 | 0.2431948 | 0.8748804 |
| CD40LG | -0.0847718 | 0.61788649 | -0.3978624 | 0.24600436 | 0.86197715 |
| PSEN1 | -0.0878906 | 0.58968827 | -0.3887563 | 0.2299135 | 0.83293468 |
| PRDX6 | -0.0969853 | 0.55161328 | -0.3965152 | 0.22120715 | 0.80090551 |
| SNAP25 | -0.100223 | 0.53834452 | -0.3992671 | 0.21809539 | 0.8004333 |
| SNCA | -0.10671 | 0.51223107 | -0.4047645 | 0.21184121 | 0.78219068 |
| VCAM1 | -0.1084684 | 0.50526343 | -0.406251 | 0.21014137 | 0.7821201 |
| CD63 | -0.1117176 | 0.49251619 | -0.4089935 | 0.2069954 | 0.7729768 |
| SOD1 | -0.1174596 | 0.4704005 | -0.4138271 | 0.20141964 | 0.75711753 |
| SLIT2 | -0.120725 | 0.45806278 | -0.4165684 | 0.19823958 | 0.75016078 |
| UCHL1 | -0.1328056 | 0.41396834 | -0.4266638 | 0.18641576 | 0.74251465 |
| RUVBL2 | -0.1350276 | 0.40612963 | -0.4285127 | 0.18423093 | 0.740204 |
| TAFA5 | -0.1559949 | 0.33644421 | -0.4458392 | 0.16345752 | 0.67406561 |
| IL4 | -0.1657926 | 0.30658581 | -0.4538619 | 0.15365222 | 0.66623455 |
| ANXA5 | -0.1681417 | 0.29968596 | -0.4557784 | 0.15129198 | 0.6648818 |
| FLT1 | -0.1706335 | 0.29247636 | -0.4578085 | 0.14878434 | 0.6648818 |
| FGF2 | -0.1791054 | 0.26880736 | -0.4646884 | 0.1402277 | 0.6648818 |
| TIMP3 | -0.179961 | 0.26648924 | -0.4653813 | 0.13936086 | 0.6648818 |
| BASP1 | -0.2150175 | 0.18272411 | -0.493474 | 0.10341884 | 0.58993785 |
| FABP3 | -0.220269 | 0.17201089 | -0.4976329 | 0.09796209 | 0.58445927 |
| HBA1 | -0.2484125 | 0.19381406 | -0.563605 | 0.12992181 | 0.6083608 |
| PARK7 | -0.2515863 | 0.11734305 | -0.5221722 | 0.06501867 | 0.45723328 |
| CXCL1 | -0.2534373 | 0.11458029 | -0.5236088 | 0.06304967 | 0.45723328 |
| GOT1 | -0.2699765 | 0.09203026 | -0.5363765 | 0.04534648 | 0.41597679 |
| YWHAZ | -0.2799854 | 0.08014952 | -0.5440443 | 0.03453609 | 0.37737066 |
| IGF1 | -0.2957694 | 0.06388339 | -0.5560473 | 0.01733734 | 0.32812831 |
| PGK1 | -0.3207566 | 0.04359277 | -0.5748297 | -0.0102738 | 0.25926224 |
| MDH1 | -0.3508472 | 0.02644056 | -0.5970983 | -0.0441647 | 0.17575197 |
| CRP | -0.8875516 | 2.34E-14 | -0.9393491 | -0.7961669 | 2.64E-12 |

**Figure S1.** Plasma proteins passing multiple testing correction in biologically determined AD compared to non-AD (Cohort 1).

**Figure S2.** Nominally significant plasma proteins in biologically determined AD compared to non-AD (Cohort 1).

**Figure S3.** Serum proteins passing multiple testing correction in biologically determined AD compared to non-AD (Cohort 1).

**Figure S4.** Nominally significant serum proteins in biologically determined AD compared to non-AD (Cohort 1).

**Figure S5.** CSF proteins passing multiple testing correction in biologically determined AD compared to non-AD (cohort 1).

**Figure S6**. Nominally significant CSF proteins in biologically determined AD compared to non-AD (cohort 1).

**Figure S7**. Comparison between NULISAseq plasma pTau217 and other assays.

**Figure S8.** Plasma proteins passing multiple testing correction in MCI Aβ+ compared to MCI Aβ- (Cohort 2).

**Figure S9.** Nominally significant plasma proteins in MCI Aβ+ compared to MCI Aβ-.

**Figure S10.** Plasma proteins passing multiple testing correction in LB+ compared to AD.

**Figure S11.** Nominally significant plasma proteins in LB+ compared to AD.

**Figure S12.** Plasma proteins of interest compared between LB+ and AD.

**Figure S13.** Plasma proteins passing multiple testing correction in *GRN*+ compared to *GRN*-.

**Figure S14.** Plasma proteins of interest compared between *GRN*+ and *GRN*-.

**Figure S15** - Correlation of the same protein targets in plasma and serum (ranked by correlation coefficient). The bars indicate the confidence intervals (CI) at 95%.

**Figure S16** - Correlation of the same protein targets in CSF and plasma (ranked by correlation coefficient). The bars indicate the confidence intervals (CI) at 95%.

**Figure S17** - Correlation of the same protein targets in CSF and serum (ranked by correlation coefficient). The bars indicate the confidence intervals (CI) at 95%.

**Figure S18** – The NPQ ratio between plasma and serum to compare abundance
